# ITGBL1 promotes cell migration and invasion through stimulating the TGF‐β signalling pathway in hepatocellular carcinoma

**DOI:** 10.1111/cpr.12836

**Published:** 2020-06-14

**Authors:** Wei Huang, Demin Yu, Mingjie Wang, Yue Han, Junyu Lin, Dong Wei, Jialin Cai, Bin Li, Peizhan Chen, Xinxin Zhang

**Affiliations:** ^1^ Research Laboratory of Clinical Virology Ruijin Hospital School of Medicine Shanghai Jiaotong University Shanghai China; ^2^ Clinical Research Center Ruijin Hospital North School of Medicine Shanghai Jiao Tong University Shanghai China; ^3^ Biliary Tract Surgery Department I Eastern Hepatobiliary Surgery Hospital Secondary Military Medical University Shanghai China

**Keywords:** hepatocellular carcinoma, invasion, ITGBL1, migration, TGF‐β

## Abstract

**Objectives:**

Integrin beta‐like 1 (ITGBL1) is involved in the migration and invasion of several cancers; however, its roles in the development and progression of hepatocellular carcinoma (HCC) remain largely unknown.

**Materials and methods:**

Immunohistochemistry staining was used to investigate the expression pattern of ITGBL1 and its prognostic values in HCC patients. The transwell, wound‐healing assays, xenograft and orthotopic mouse models were employed to determine the effects of ITGBL1 on HCC cell migration and invasion in vitro and in vivo. The biological mechanisms involved in cell migration and invasion caused by ITGBL1 were determined with Western blotting and RT‐PCR methods.

**Results:**

ITGBL1 expression was significantly increased in HCC tissues compared to adjacent normal tissues. Patients with higher ITGBL1 expression were associated with more reduced overall survival. ITGBL1 overexpression promoted migration and invasion in SMMC‐7721 and HepG2 cells in vitro and in vivo, whereas knockdown or knockout ITGBL1 in CSQT‐2 cells significantly reduced cell migration and invasion abilities. In SMMC‐7721 cells, ITGBL1 overexpression stimulated TGF‐β/Smads signalling pathway, along with the KRT17 and genes involved in the epithelial‐mesenchymal transition (EMT). In contrast, ITGBL1 knockout inhibited the TGF‐β/Smads signalling pathway in CSQT‐2 cells.

**Conclusions:**

These findings suggested that ITGBL1 promoted migration and invasion in HCC cells by stimulating the TGF‐β/Smads signalling pathway. ITGBL1 could be a promising prognostic biomarker, as well as a potential therapeutic target in HCC.

## 1 INTRODUCTION

Liver cancer ranks as the second leading cause of cancer‐related mortalities worldwide, which led to an estimated 810 000 deaths per year.^1^ Importantly, 90% of primary liver cancer patients have been diagnosed as hepatocellular carcinoma (HCC).^2^ Various risk factors of HCC have been identified, including hepatitis B virus (HBV) or hepatitis C virus (HCV) infection, exposure to aflatoxin B1 (AFB1) and/or aristolochic acid and a history of diabetes, etc^2^; these factors have provided efficient targets for HCC prevention. The prognosis of HCC patients is usually poor, with the 5‐year post‐operative survival rate being 25%‐50% for patients with early‐stage HCC.^3^ However, according to the Surveillance, Epidemiology and End Results (SEER) database,^4^, ^5^ the 5‐year survival rate of HCC patients with distant metastases was dropped below 5%. After surgery, about 70% of patients have been shown to relapse or develop distant metastasis within 2 years.^3^ Thus, identification of novel therapeutic targets or prognosis prediction biomarkers may provide clues for the development of novel treatment strategies.

ITGBL1 (integrin beta‐like 1, also termed as OSCP or TIED) encodes a beta integrin‐related protein that is a member of the epidermal growth factor (EGF)‐like protein family. ITGBL1 was first cloned and characterized in 1998 from the overlapping cDNA clones of foetal lung, human umbilical vein endothelial cell (HUVEC) and osteoblast cDNA libraries.^6^ Previous studies also showed that ITGBL1 was involved in the development and progression of several cancers. In breast cancer tissues, ITGBL1 is co‐expressed with bone remodelling‐ and bone metastasis‐related genes,^7^ and it could promote the bone metastasis of breast cancer cells through activating the TGF‐β signalling pathway.^8^ In colorectal cancer (CRC), the expression of ITGBL1 was upregulated,^9^, ^10^ and the primary tumours could release ITGBL1‐rich extracellular vesicles to induce the activation of resident fibroblasts in remote organs, which promotes the metastatic cancer growth.^11^ In our previous gene expression profiling data set of HBV‐related liver fibrosis tissues, ITGBL1 was positively associated with the fibrosis stage and identified as a key regulator of fibrogenesis in patients with HBV infection.^12^ In addition, ITGBL1, together with CD24, CXCL6, EHF, LUM and SOX9, has been suggested to act as a predictive biomarker of cirrhosis in patients with chronic HBV infection.^13^ Whether ITGBL1 is involved in the pathogenesis and progression of HCC and the related underlying mechanisms remain unclear.

In the current study, we aimed to determine the gene expression profiling of ITGBL1 and its prognostic values in HCC patients. The roles of ITGBL1 in HCC cell migration and invasion and the corresponding underlying molecular mechanisms were also determined. This study may unveil the new insights into the progression of HCC and provide novel prognosis biomarkers or potential therapeutic targets for HCC patients.

## 2 MATERIALS AND METHODS

### 2.1 HCC patient recruitment

The formalin‐fixed, paraffin‐embedded (FFPE) tumour and adjacent normal tissue samples from 98 HCC patients, who received the curative surgery treatment in the Eastern Hepatobiliary Surgery Hospital of the Second Military Medical University between July 2012 and February 2014, were collected.^14^ The patients who did not receive any anti‐cancer treatments before they underwent curative resection surgery were included in this study. If the patients who had a distant metastasis or (and) a history of other malignancies were excluded in the study. And if the patients who had received the palliative treatments or (and) the liver transplant treatment were also been excluded in the study. Patients were also excluded if they were unwilling to participate in the study. Based on the pathological examination, all patients were diagnosed with HCC. Personal information and clinicopathological parameters of the patients were extracted and assessed by the clinicians from the medical records. The results of the preoperative biochemical tests and imaging examination of patients were also retrieved from the medical records. The follow‐ up of patients was performed by checking the medical records or by telephone calls at half‐year intervals, and the last follow‐up was completed in September 2016. Sufficient follow‐up data were available for 60 patients. Besides, another 22 pairs of fresh tumour and adjacent non‐tumour tissues of the HCC patients were frozen and stored in liquid nitrogen directly until use. For each patient, the written informed consent was obtained by individual communications and the study protocol has been approved by the ethics committees and the institutional review board of the Second Military Medical University.

### 2.2 Western blotting analysis

The proteins from the liver tissues and cells were extracted using the cell lysis buffer (cat. no. 9803S; Cell Signaling Technology, inc.) containing protease inhibitor cocktail (cat. no. P8340; Sigma‐Aldrich) and phosphatase inhibitor cocktails (cat. no. P2850 and P5726; Sigma‐Aldrich). The BCA protein assay kit (cat. no. 23225; Thermo Fisher Scientific) was used to measure the protein concentrations according to the manufacturer's guidelines. Equal amounts of the total protein samples (20 μg) were separated via SDS‐PAGE on 8%‐12% polyacrylamide gels. The resultant protein bands were transferred onto polyvinylidene fluoride (PVDF) membranes (cat. no. IEVH00005; Merck) for 60 minutes. Next, the membranes were blocked with 5% skimmed milk for 2 hours at room temperature (cat. no. 232100; BD), washed three times with 1 × PBST and then incubated with the primary antibodies overnight at 4°C. After reheated for 0.5 hours at room temperature, the membranes were washed three times with 1 × PBST and then incubated with the secondary antibodies, that is horseradish peroxidase‐linked IgG, for another 2 hours at room temperature. After washed three times and 10 minutes each time, the signal intensities of the membranes were visualized on the Tanon‐5200 Chemiluminescent Imaging System (Tanon). All experiments were performed at least three independent times to obtain consistent results.

The primary antibodies for anti‐ITGBL1 (1:1,000; cat. no. ab93592) and anti‐Cytokeratin 17 (1:1,000; cat. no. ab53707) for Western blotting were purchased from Abcam. The primary antibodies for rabbit monoclonal anti‐GAPDH (1:5,000; cat. no. 5174), rabbit anti‐N‐cadherin (1:1000; cat. no.13116), rabbit anti‐Vimentin (1:1000; cat. no.5741), rabbit anti‐Claudin‐1 (1:1000; cat. no.13255), rabbit anti‐Snail (1:1000; cat. no.3879), rabbit anti‐Slug (1:1000; cat. no.9585), rabbit anti‐β‐catenin (1:1000; cat. no.8480), rabbit anti‐Smad2 (1:1000; cat. no.5339), rabbit anti‐Phospho‐Smad2 (1:1000; cat. no.3108), and the secondary antibody used was horseradish peroxidase‐linked IgG (1:5000; cat. no.7074) were provided by Cell Signaling Technology, Inc.

### 2.3 Immunohistochemistry (IHC) staining

Tissue microarrays (TMAs) of 98 HCC patients were constructed as reported previously.^14^ IHC staining was performed to detect the expression of ITGBL1 for each patient. The TMAs were dewaxed twice with xylene for 20 minutes each time and rehydrated with gradient ethanol solutions for 5 minutes at a time. After reheated for 0.5 hours at room temperature, the TMAs were then treated with 3% hydrogen peroxide in methanol to quench the endogenous peroxidase activity for 30 minutes at room temperature. Then, the TMAs were boiled with the citric acid solution (pH = 6.0) for antigen retrieval at 92°C for 40 minutes. After natural cooling, the TMAs were washed with 1 × PBS and then blocked using 1% foetal bovine serum (FBS) for 30 min at room temperature. And the TMAs were incubated with anti‐ITGBL1 antibodies (1:100; cat. no. NBP1‐82473; Novus Biologicals) at 4°C overnight. Then, the TMAs were washed with 1 × PBS for three times and incubated with peroxidase polymer‐labelled goat anti‐rabbit secondary antibodies (cat. no. HAF008; Novus Biologicals) at room temperature for 1 hour. The TMAs were washed three times with 1 × PBS and then incubated with diaminobenzidine, counter‐stained with haematoxylin, dehydrated and cover‐slipped. The TMAs were examined using a Leica CTR5000 microscope (Leica Microsystems).

After hiding the clinical and pathological information of the HCC patients, all the stained sections were evaluated independently by two pathologists. The expression of ITGBL1 was assessed by measuring the staining intensity and the percentage of the positively stained cells. The staining intensity was categorized as 0 (negative staining), 1 (weak staining), 2 (moderate staining) and 3 (strong staining). The percentages of positive cells were grouped as 1 (0%‐25%), 2 (25%‐50%), 3 (50%‐75%) and 4 (>75%). The immunoreactivity score (IRS) was calculated by multiplying the staining intensity with the percentage of positively stained cells.^15^ The patients of HCC patients were divided into two groups according to the median IRS score of ITGBL1 expression level in cancer tissues (higher vs lower).

### 2.4 Cell cultures and lentivirus infection

Human hepatoma cell lines (HepG2, Huh7, Hep3B, SMMC‐7721, Bel‐7404, CSQT‐2, and MHCC‐97H), immortal hepatic LO2 cells and HEK293FT cells were obtained from the Ruijin Hospital, Shanghai Jiao Tong University School of Medicine. These cells were cultured in high‐glucose Dulbecco's modified Eagle's medium (DMEM; cat. no. 10569010; Thermo Fisher Scientific) containing with 10% foetal bovine serum (FBS; cat. no. 10099141; Thermo Fisher Scientific) and 100 U/mL penicillin‐streptomycin (cat. no. 10378016; Thermo Fisher Scientific) in an incubator under 5% CO_2_ conditions at 37°C.

The lentiviral overexpression plasmids carrying the ITGBL1 open reading frame (ORF) and negative control plasmids were purchased from GeneChem. SMMC‐7721 and HepG2 cells were seeded into 6‐well cell culture plates. When cells grew to 70%‐80% confluence, the cells were infected with the lentiviruses along with 8 µg/mL polybrene (cat. no. TR‐1003‐G; Merck) for 12 hours. Forty‐eight hours later, the lentiviruses were removed and the cells were cultured for another 48 hours. The infected cells were treated with 3 µg/mL puromycin (cat. no. A1113803; Thermo Fisher Scientific) for 48 hours to select the positive clones. The overexpression of ITGBL1 was confirmed by real‐time polymerase chain reaction (RT‐PCR) and Western blotting analyses. LY2109761, a selective TGF‐β receptor type I/II (TGFBRI/II) dual inhibitor, was purchased from Selleckchem (cat. no. S2704; Selleck Chemicals).

### 2.5 Construction of a CRISPR/Cas9 lentivirus vector targeting ITGBL1

The sgRNA sequences of ITGBL1 were designed using the online CRISPR Design Tool (http:/tools.genome‐engineering.org), and three different sgRNA1, sgRNA2 and sgRNA3 were selected (Table S2).^16^ Synthetic sgRNA oligonucleotides were cloned into the lentiCRISPRv2 construct at BsmBI restriction sites (cat. no. 98290; Addgene). The lentivirus was constructed by co‐transfecting with the lentiCRISPRv2‐sgRNA vector and three packaging vectors (pmDlg, Rev and pVSVg) into HEK293FT cells using Lipofectamine 2000 reagent (cat. no. 11668019; Thermo Fisher Scientific). LentiCRISPRv2 without sgRNA was used as the negative control (Lv‐control). CSQT‐2 cells were infected with the CRISPR/Cas9 lentiviruses or the negative control, and the positive clones were selected using puromycin (6 μg/mL) for 48 hours. The puromycin‐selected cells were subjected to monoclonal screening and the gene knockout of ITGBL1 was confirmed by Sanger sequencing and Western blotting analyses. The primer sequences used for PCR amplification and sgRNA sequences are listed in Table S2.

### 2.6 RNA extraction and real‐time PCR

The TRIzol reagent (cat. no. 16 096 040; Thermo Fisher Scientific) was used to extract the total RNAs from the liver tissue specimens or cells following the manufacturer's instructions. The reverse transcription and RT‐PCR were performed using the PrimeScript™RT Master Mix (cat. no. RR036A; Takara) and the QuantiNova SYBR Green PCR Kit (cat. no. 208054; Qiagen), respectively. The amplification parameters were 95°C for 2 minutes, followed by 40 cycles of 95°C for 5 seconds and 60°C for 10 seconds, according to the manufacturer's protocol. The mRNA level of GAPDH was used as an internal control. The gene‐specific RT‐PCR primers used in this study are provided in Table S3. The relative expression of the mRNA levels was calculated using the ΔΔCt (2^−ΔΔCT^) method.^17^

### 2.7 Wound‐healing assay

SMMC‐7721, HepG2 and CSQT‐2 cells were plated into 6‐well culture plates at a density of 5 × 10^5^ cells/well and grown until confluence overnight. After scratching three parallel lines onto the confluent cell layer, the cells were washed three times with 1 × PBS and culture with DMEM medium containing 10% FBS. Images of migrating cells were sequentially taken 0, 24 and 48 hours after the scratch. The relative wound region among different sample groups was evaluated and compared.

### 2.8 Transwell assay

The transwell assays were performed using the transwell chamber (pore size of 8.0 µm; Corning) to evaluate the cellular migration and invasion abilities. For invasion assays, 500 μL of DMEM medium without FBS was placed into the upper chambers to rehydrate the Matrigel for 2 hours at 37°C. After removing the supernatants, a total of 5 × 10^4^ cells containing DMEM medium without FBS were seeded into the upper chambers, and 600 µL of DMEM medium with 10% FBS was added to the bottom chambers of 24‐well plates. After incubation for 24 hours, the cells in the upper chambers were fixed with 4% paraformaldehyde for 15 minutes and then stained with crystal violet for 5 minutes. The cells into the upper chambers were swabbed using cotton swabs. The numbers of migrating or invading cells were counted in three randomly selected fields.

### 2.9 The xenograft and metastasis mice models

Athymic male BALB/c nude mice were used to investigate the effects of ITGBL1 on the tumour growth‐ and metastasis‐promoting abilities of SMMC‐7721 and CSQT‐2 cells. To establish the xenograft model, a total of 2 × 10^6^ SMMC‐7721 cells in 100 μL of serum‐free DMEM medium containing 50% Matrigel were subcutaneously inoculated into the rear right groin flank of the mouse (6 weeks old, N = 7 for each group), and measured the length (*L*) and width (*W*) of the tumour every three days. The tumour volume was calculated with the equation 1/2 × *L *× *W* ^2^. The mice were sacrificed 34 days after the injection and the tumour weights were measured by haematoxylin and eosin (H&E) staining.

To establish the lung metastasis model, a total of 1 × 10^6^ SMMC‐7721 or CSQT‐2 cells in 100 µL of DMEM medium without FBS were injected through the tail veins of male nude mice (5 weeks old, N = 8 or 9 for each group as indicated). Four weeks later, the mice were euthanized, and the histopathological manifestations of pulmonary metastases in the mouse model were observed by H&E staining.

To establish the orthotopic model, 1 × 10^6^ CSQT‐2 cells in 50 μL of DMEM medium without FBS were injected into the left liver lobe of each mouse (5 weeks old, N = 8 for each group). The mice were sacrificed 6 weeks after the injection and the number of metastatic nodules in their lungs was counted by H&E staining. All the animal experiments were approved by the animal experimentation ethics committee of Ruijin Hospital, affiliated to the Shanghai Jiaotong University School of Medicine.

### 2.10 RNA‐sequencing analysis

Total RNA from the ITGBL1‐overexpressing and control SMMC‐7721 cells were isolated and quantified. The Qubit^®^ RNA Assay Kit on a Qubit^®^2.0 Fluorometer (Thermo Fisher Scientific) was used to measure the concentrations of RNA, and the RNA Nano 6000 Assay Kit of the Bioanalyzer 2100 system (Agilent Technologies) was used to assess the integrity of RNA. The sequencing libraries were generated using the NEBNext^®^ UltraTM RNA Library Prep Kit for Illumina^®^ (NEB), following the manufacturer's recommendations. The differentially expressed genes between the two groups’ cells were analysed using the DESeq2 R package (1.16.1). Genes with a false‐discovery rate (FDR) adjusted *P*‐value of <.05, as estimated using the DESeq2 package, were assigned as differentially expressed genes. The differentially expressed genes between the conditions are listed as Table S4.

### 2.11 Statistical analysis

The Oncomine database (https://www.oncomine.org) and The Cancer Genome Atlas (TCGA) HCC data set were used to evaluate the mRNA expression levels of ITGBL1 and KRT17 in the normal and cancer tissues of HCC patients, and compared using Student's *t* test between the groups. The non‐parametric Wilcoxon's rank test was used to compare the protein expression of ITGBL1 in the tumour tissues and the matched non‐tumour tissues. The *χ* ^2^ test was performed to evaluate the correlations between the ITGBL1 expression and clinicopathological features. The Kaplan‐Meier plot, together with the log‐rank test, was used to compare the overall survival (OS) between the groups. Univariate Cox regression was used to determine the associations between the clinical features and OS of the patients. To determine the independent prognostic factors associated with the OS of the HCC patients, the multivariate Cox proportional hazards model was applied. One‐way analysis of variance (ANOVA) was used to test the overall differences between the groups, and the difference between subgroups was compared by Turkey's honestly significant difference (Turkey's HSD) test. The SPSS (version 22.0) and the GraphPad Prism software (version 6.0, GraphPad) were used for the statistical analyses. Two‐sided *P*‐value < .05 was considered as statistically significant for all analyses.

## 3 RESULTS

### 3.1 ITGBL1 expression is upregulated in HCC tumour tissues

**FIGURE 1:**
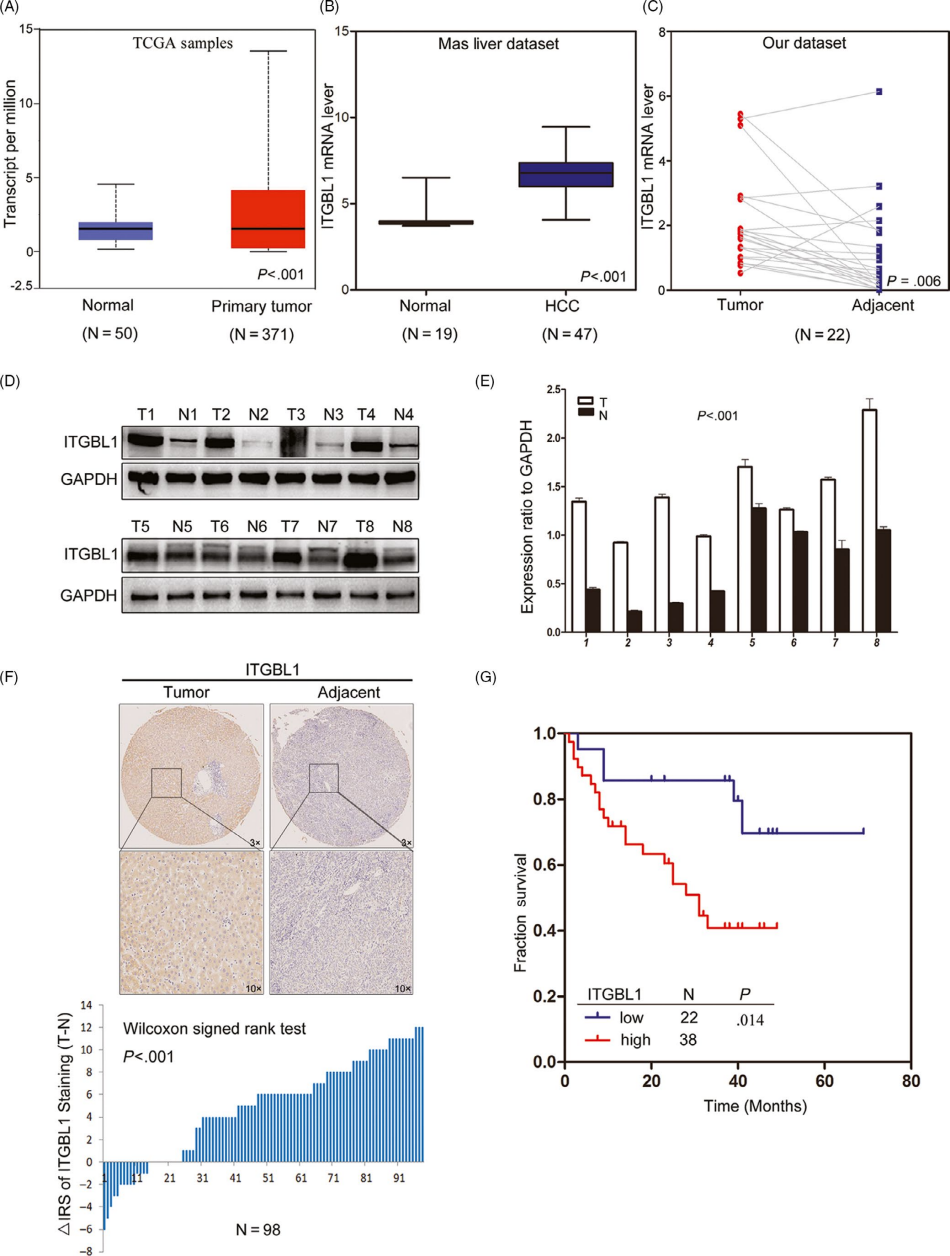
ITGBL1 expression is upregulated in HCC tissues and associated with the prognosis of HCC patients. A, ITGBL1 expression in the normal liver tissues (n = 50) and primary liver tumour tissues (n = 371), as revealed by the TCGA HCC data set (*P* < .001). B, ITGBL1 expression in the normal liver (n = 19) and HCC tissues (n = 47), as revealed by the Oncomine database (GSE14323; *P* < .001). C, Different ITGBL1 mRNA expression levels in 22 paired samples of HCC tumour tissues and adjacent non‐cancerous tissues, as determined by RT‐PCR analyses (*P* = .0057). D, Western blotting analyses of the ITGBL1 protein expression levels in eight randomly selected tumour tissues and the matched adjacent non‐tumour tissues from HCC patients; GAPDH was used as the internal loading control. E, Greyscale analysis of the ITGBL1 expression level, compared to the GAPDH expression level, in the eight patients (*P* < .001). T, tumour tissue; N, normal tissue. F, Immunochemistry staining of ITGBL1 in the TMAs with HCC tissues and adjacent non‐cancerous tissues (n = 98; *P* < .001). G, The Kaplan‐Meier plot for the overall survival of HCC patients according to ITGBL1 expression levels (higher vs lower, log‐rank test, *P* = .014)

To determine the expression pattern of ITGBL1 in HCC tissues, the TCGA and Oncomine databases were searched, and we found that the ITGBL1 mRNA levels were significantly increased in HCC cancer tissues compared to the adjacent normal tissues (Figure 1A,B). We confirmed these results in 22 pairs of HCC patients by the RT‐PCR method (Figure 1C). Further, the Western blotting results also suggested that the protein expression level of ITGBL1 was upregulated in eight paired HCC tumour tissues, compared with the adjacent non‐tumour tissues (Figure 1D,E). Using the IHC staining method, we investigated the ITGBL1 expression level in 98 paired HCC tissues. We found that ITGBL1 was highly expressed in HCC tumour tissues, whereas weak or negative expression of ITGBL1 was observed in the adjacent non‐tumour tissues (Wilcoxon's signed‐rank test, *P* < .001, Figure 1F). These results implicated that both the mRNA and protein expression level of ITGBL1 were significantly increased in HCC tumour tissues compared to adjacent normal tissues.

### 3.2 Higher ITGBL1 expression predicts a poorer prognosis in HCC patients

Next, we evaluated the associations of ITGBL1 protein expression levels and the clinicopathological characteristics in 98 HCC patients. We found that ITGBL1 expression level was significantly associated with the tumour encapsulation status (*P* = .037), but not other clinicopathological characteristics (Table S1). The Kaplan‐Meier survival analysis suggested that HCC patients with higher ITGBL1 expression had relatively poorer OS than those with lower ITGBL1 expression (log‐rank test, *P* = .014, Figure 1G). In the univariate analysis, the clinicopathological characteristics including AFP (*P* = .038), tumour number (*P* < .001), BCLC stage (*P* < .001), vascular invasion (*P* = .003) and ITGBL1 expression (*P* = .014) were significantly associated with the OS in HCC patients (Table 1). The multivariate Cox proportional hazards model also suggested that ITGBL1 was an independent prognostic factor for HCC patients (*P* = .040; Table 1). These results highlighted that patients with relatively higher ITGBL1 expression level were associated with the poorer OS; the ITGBL1 expression level may, thus, serve as a novel prognostic factor for HCC patients.

**TABLE 1:** Univariate and Multivariable analysis of clinic‐pathologic factors for overall survival in 60 patients with HCC

Risk factors	Univariate analysis			Multivariable analysis		
	HR	95% CI	*P*	HR	95% CI	*P*
AFP, ng/mL (<400 vs ≥400)	0.421	0.186‐0.954	.038	0.457	0.152‐1.376	.164
Tumour number (Singer vs Multiple)	0.045	0.010‐0.207	<.001	0.612	0.173‐2.164	.446
BCLC (0/A vs B/C)	0.131	0.050‐0.341	<.001	0.211	0.035‐1.284	.091
Vascular invasion (Positive vs Negative)	0.226	0.085‐0.602	.003	1.695	0.371‐7.742	.496
ITGBL1 (High vs Low)	0.370	0.168‐0.817	.014	2.883	1.050‐7.910	.040
Gender (Male vs Female)	0.746	0.255‐2.18	.592			
Age (<50 vs ≥50)	1.287	0.587‐2.823	.529			
HbsAg (Negative vs Positive)	0.856	0.308‐2.379	.766			
Cirrhosis (Negative vs Positive)	0.689	0.308‐1.540	.364			
Tumour size, cm (<5 vs ≥5)	0.444	0.194‐1.017	.055			
Tumour differentiation (I/Ⅱ vs Ⅲ/Ⅳ)	0.459	0.173‐1.219	.118			
Tumour encapsulation (Non‐intact vs Intact)	1.399	0.630‐3.108	.410			

Abbreviations: 95% CI, 95% confidential interval; AFP, α‐fetoprotein; BCLC, Barcelona Clinic Liver Cancer; HR, hazard ratio.

### 3.3 Overexpression of ITGBL1 promotes HCC cell migration and invasion in vitro and in vivo

**FIGURE 2:**
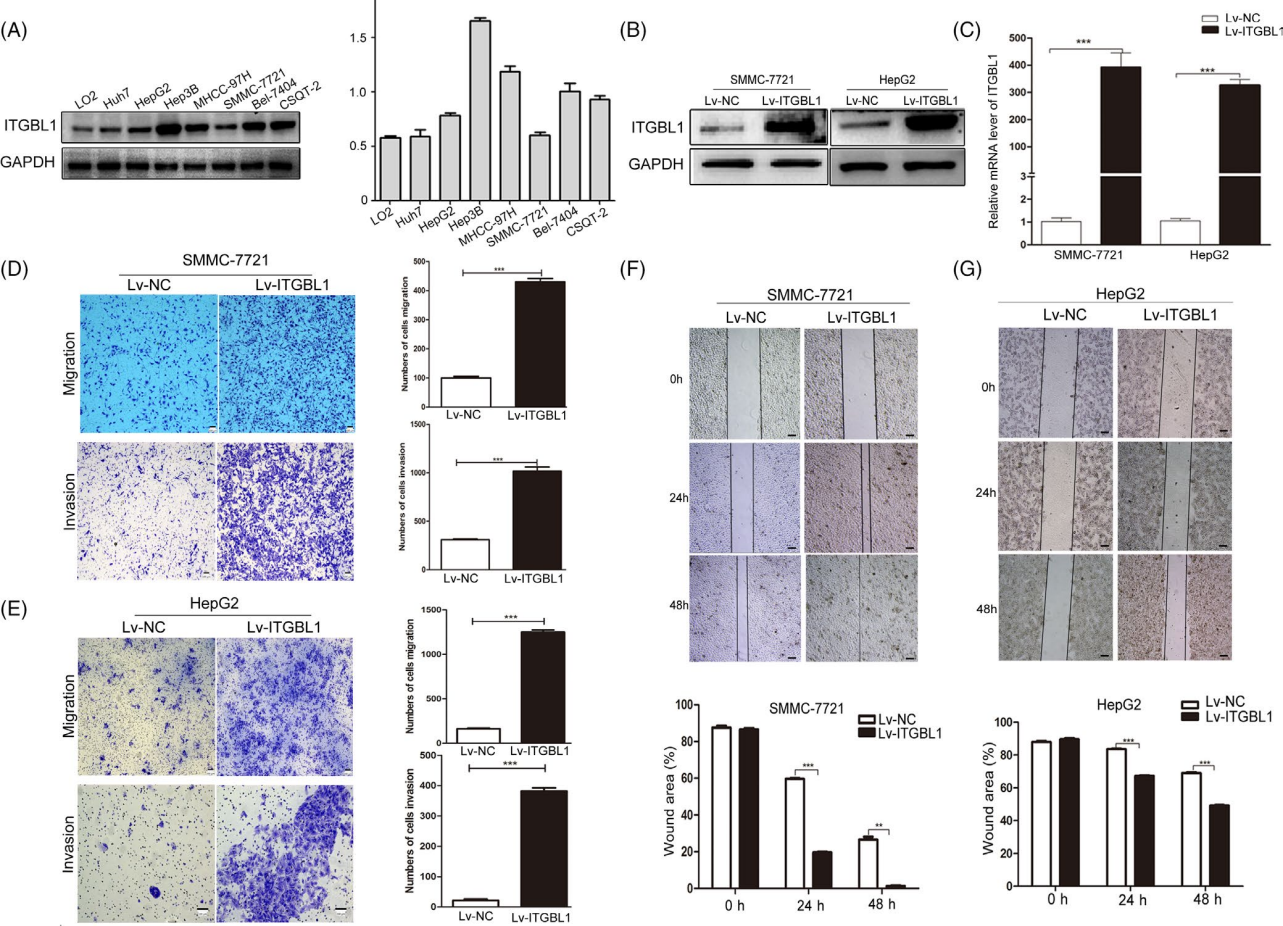
ITGBL1 overexpression promotes HCC cell migration and invasion in vitro. A, Western blotting analysis of ITGBL1 expression in HCC cell lines and the normal LO2 hepatocytes. Western blotting (B) and RT‐PCR analyses (C) for the expression of ITGBL1 in SMMC‐7721 cells and HepG2 cells infected with the ITGBL1‐overexpression or mock vector lentivirus. The migration and invasion assays were performed to investigate the migratory and invasive abilities of SMMC‐7721 (D) and HepG2 (E) cells with or without ITGBL1 overexpression. Wound‐healing assays were performed to determine the migratory abilities of SMMC‐7721 cells (F) and HepG2 cells (G) with or without ITGBL1 overexpression. ^**^ *P* < .01 and ^***^ *P* < .001 indicate a significant difference between the groups

To investigate the roles of ITGBL1 in HCC cells, we first evaluated the expression of ITGBL1 in several liver cell lines. As shown in Figure 2A, the expression of ITGBL1 was higher in HCC cell lines, including Huh7, HepG2, Hep3B, SMMC‐7721, Bel‐7404, CSQT‐2 and MHCC‐97H cells, compared to the normal LO2 hepatocytes. We stably upregulated the ITGBL1 expression in the SMMC‐7721 cells and HepG2 cells using a lentivirus system, which was confirmed by RT‐PCR and Western blotting analyses (Figure 2B,C). The transwell migration and invasion assays in together with the wound‐healing assays showed that ITGBL1 overexpression significantly enhanced the migration and invasion abilities of SMMC‐7721 cells and HepG2 cells in vitro (Figure 2D‐G). Furthermore, we also demonstrated the roles of ITGBL1 in HCC cell migration with SK‐Hep1 cells (Figure S1). ITGBL1 overexpression also promotes SK‐Hep1 cells migration in vitro (Figure S1B,C).

**FIGURE 3:**
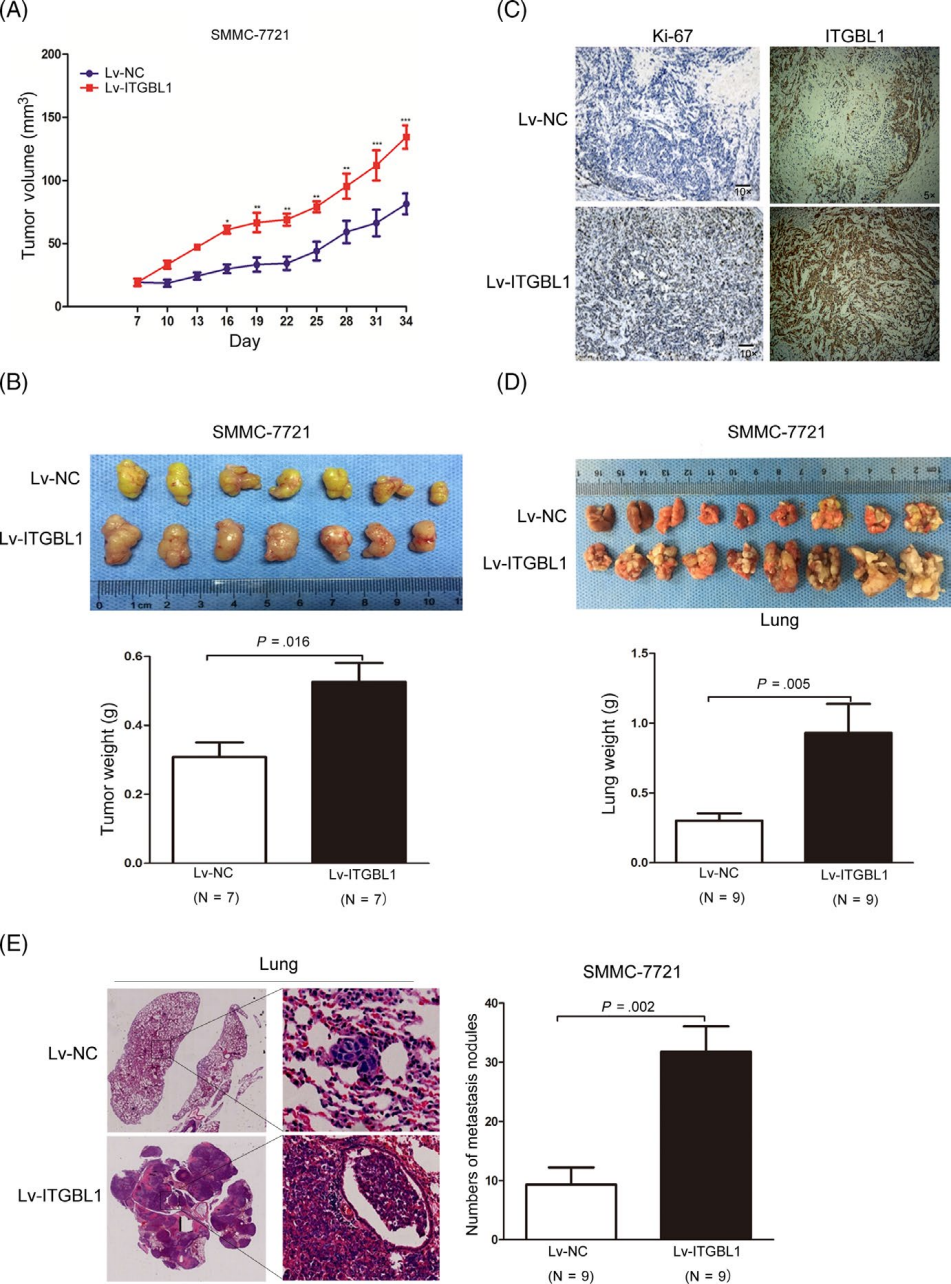
ITGBL1 overexpression promotes HCC cell proliferation and migration in vivo. A, SMMC‐7721 cells infected with ITGBL1 overexpression lentivirus (Lv‐ITGBL1) or control mock lentivirus (Lv‐control) were subcutaneously injected in the male nude mice (N = 7 for each group). The tumour volumes were measured every three days after the injection. Data are shown as the mean ± SD. B, Representative images (upper panel) of the tumour tissues thirty‐four days after the injection of the cells as described in (A). The tumour weights were measured and compared (lower panel, *P* = .016). Data are shown as the mean ± SD. C, The xenograft tumour sections were subjected to IHC staining using antibodies against ITGBL1 and Ki‐67. D, SMMC‐7721 cells infected with ITGBL1 overexpression lentivirus (Lv‐ITGBL1) or control mock lentivirus (Lv‐control) were injected into the nude mice through the tail vein (N = 9 for each group). Lungs from the mice in each group are shown 60 days after the injection (upper panel); the lung weights were measured and compared (lower panel, *P* = .005). E, Representative image of the H&E‐stained lung metastasis nodules (left) following the injection of the cells described in (D) via the tail vein. The numbers of tumour nodules in the lungs were counted and compared (right, *P* = .002). The data are shown as the mean ± SD. ^*^ *P* < .05, ^**^ *P* < .01 and ^***^ *P* < .001 indicate a significant difference between the groups

Subsequently, to confirm the effects of ITGBL1 on HCC tumour growth in vivo, SMMC‐7721 cells with or without stable ITGBL1 overexpression were subcutaneously injected into the nude mice and the tumour size of the xenografts were measured. Strikingly, we found that the SMMC‐7721 cells with ITGBL1 overexpression significantly enhanced the tumour growth abilities compared to the cells from the control group (Figure 3A). Thirty‐four days after the injection, the tumour weight of the Lv‐ITGBL1‐transfected SMMC‐7721 cells were markedly higher than the Lv‐control‐transfected cells in the mice (Figure 3B). The Ki‐67 staining of the xenografts suggested that the tumour with the ITGBL1‐overexpressing SMMC‐7721 cells had higher proliferation abilities than the control SMMC‐7721 cells (Figure 3C). We further evaluated the influences of ITGBL1 on lung metastasis of SMMC‐7721 cells via the tail vein injection models. Ninety days after the injection, we observed that ITGBL1 overexpression significantly promoted the lung metastasis abilities of SMMC‐7721 cells, compared to the control SMMC‐7721 cells (Figure 3D), and the mice injected with the ITGBL1‐overexpressing cells had more lung metastatic nodules than the control cells (Figure 3E). These data demonstrated that ITGBL1 overexpression promoted HCC cell migration and invasion in vitro and in vivo.

### 3.4 Knockout or knockdown of ITGBL1 suppresses HCC cell migration and invasion in vitro and in vivo

**FIGURE 4:**
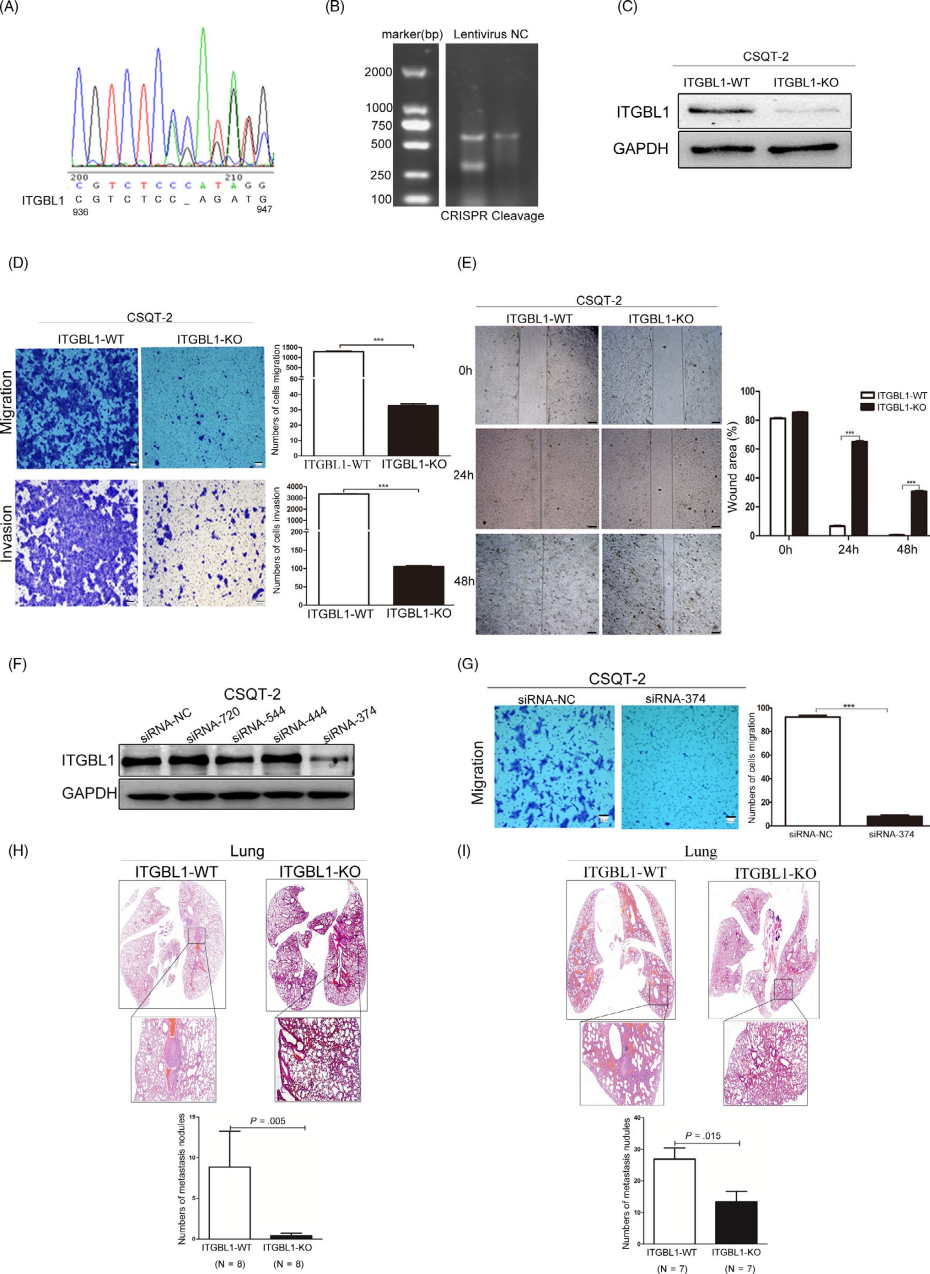
Knockout of ITGBL1 inhibits HCC cell migration and invasion in vitro and in vivo. A, Sequencing analysis of the CRISPR/Cas9‐mediated knockout of ITGBL1 gene. B, T7E1 assay results of CSQT‐2 cells infected with the control or CRISPR/Cas9‐guided ITGBL1 knockout plasmid. C, Western blotting analysis for the quantification of the ITGBL1 expression in the ITGBL1‐knockout (ITGBL1‐KO) and wild‐type (ITGBL1‐WT) CSQT‐2 cells. D, The migration (upper panel) and invasion assays (lower panel) were performed to investigate the effect of ITGBL1 on CSQT‐2 cells with or without ITGBL1 knockout. E, Wound‐healing assays were performed to investigate the migratory abilities of CSQT‐2 cells with or without ITGBL1 knockout. F, Western blotting analysis of the ITGBL1 expression in CSQT‐2 cells transfected with the siRNA targets ITGBL1 mRNA (si‐ITGBL1) or negative control (si‐NC). G, Transwell migration assays were performed to investigate the migratory abilities of CSQT‐2 cells with or without ITGBL1 knockdown. H, H&E staining of the pulmonary metastases formed in the mouse model following the injection of CSQT‐2 cells with or without ITGBL1 knockout via the tail vein (upper panel). The metastatic nodules in the lungs were counted and compared (lower panel, *P* = .005). The data are shown as the mean ± SD. I, Histopathological analysis of pulmonary metastases in the mouse orthotopic model following the injection of the CSQT‐2 cells with or without ITGBL1 knockout (upper panel); the metastatic nodules in the lungs of the mice were counted and compared (lower panel, *P* = .015). ^***^ *P* < .001 indicates a significant difference between the groups

To further verify the roles of ITGBL1 in HCC cell migration and invasion, we knocked out ITGBL1 in CSQT‐2 cells which show relatively higher ITGBL1 expression using the CRISPR/Cas9 system (Figure 4A‐C). Consistent with previous findings, the transwell and wound‐healing assays suggested that ITGBL1 knockout suppressed the migration and invasion abilities of CSQT‐2 cells in vitro (Figure 4D,E). Meanwhile, downregulating the expression of ITGBL1 by siRNA also reduced CSQT‐2 cell migration abilities (Figure 4F,G).

The tail vein injection model also suggested that ITGBL1 knockout in CSQT‐2 cells significantly reduces its lung metastasis abilities compared to wild‐type cells (Figure 4H). Besides, the orthotopic liver tumour models also showed that ITGBL1 knockout significantly inhibited the metastatic colonization of CSQT‐2 cells in the lungs, compared to the injection of control CSQT‐2 cells (Figure 4I). These data demonstrated that ITGBL1 suppression inhibited the migration and invasion of HCC cells both in vitro and in vivo.

### 3.5 ITGBL1 regulates the expression of KRT17

**FIGURE 5:**
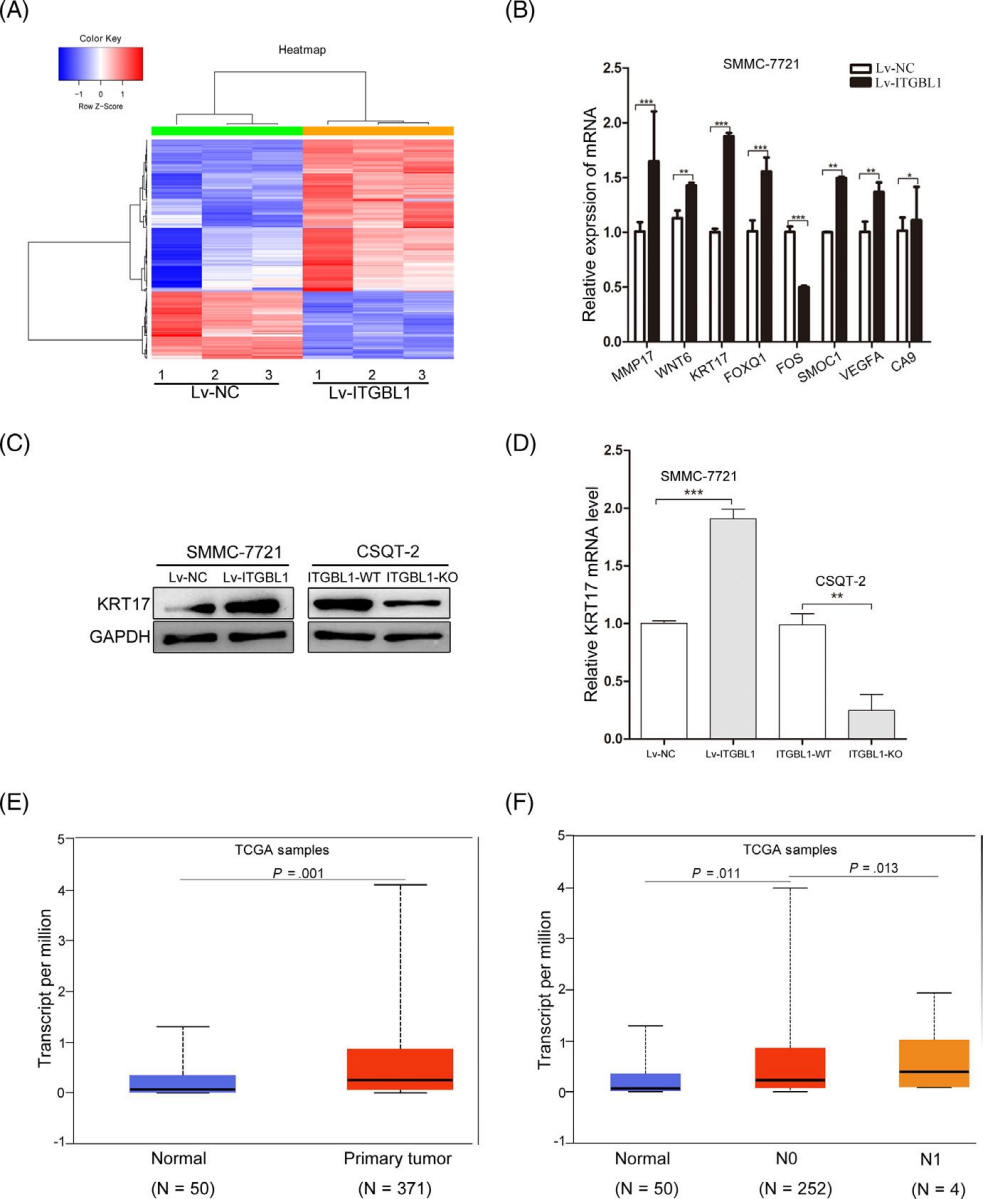
ITGBL1 regulates the expression of KRT17. A, Hierarchical clustering of 196 differentially expressed transcripts identified in RNA‐sequencing (≥1.5‐fold change and FDR P‐value < 0.05) in Lv‐ITGBL1‐transfected cells, compared to the Lv‐NC‐transfected cells (N = 3 for each group). B, Validation of the effects of ITGBL1 on the mRNA expression levels identified in RNA‐sequencing using the RT‐PCR methods in SMMC‐7721 cells. The data are expressed as the mean ± SEM. Western blotting (C) and RT‐PCR analyses (D) for the quantification of KRT17 expression levels in SMMC‐7721 cells with or without ITGBL1 overexpression and CSQT‐2 cells with or without ITGBL1 knockout. E, KRT17 expression levels in the normal liver tissues (n = 50) and primary liver tumour tissues (n = 371), as revealed by the data for HCC samples in the TCGA database. F, KRT17 expression in the normal liver tissues (n = 50), HCC tissues with no regional lymph node metastasis (N0, n = 252) and HCC tissues with 1‐3 axillary lymph nodes (N1, n = 4) were determined in TCGA database. ^*^ *P* < .05, ^**^ *P* < .01 and ^***^ *P* < .001 indicate a significant difference between the groups

To uncover the mechanisms underlying the ITGBL1‐mediated regulation of cellular metastasis and invasion, we performed the RNA‐sequencing analysis of the SMMC‐7721 cells with or without ITGBL1 overexpression. A total of 195 differentially expressed genes were identified, including 135 upregulated and 60 downregulated genes (Figure 5A and Table S4). Eight genes, including MMP17, WNT6, KRT17, FOXQ1, FOS, SMOC1, VEGFA and CA9, had been verified via the RT‐PCR method (Figure 5B). We noticed that the expression of one gene, KRT17, which has been reported to stimulate the migration and invasion of the tumour cells,^18^, ^19^ was significantly increased following ITGBL1 overexpression. The Western blotting results also confirmed that the KRT17 expression was significantly increased in the ITGBL1 overexpressing SMMC‐7721 cells, but was significantly decreased in the ITGBL1 knockout CSQT‐2 cells (Figure 5C). These results were confirmed by RT‐PCR analyses through overexpressing and knockout of ITGBL1 in SMMC‐7721 and CSQT‐2 cells, respectively (Figure 5D). Subsequently, we further determined the expression of KRT17 in the TCGA HCC data set and found that the KRT17 mRNA expression level is significantly higher in primary liver tumour tissues than the normal liver tissues (Figure 5E). Moreover, we also found that the expression of KRT17 was increased in HCC tumours with the lymph node metastasis (Figure 5F). Taken together, these results demonstrated that KRT17 might be regulated by ITGBL1 in HCC cells.

### 3.6 ITGBL1 induces EMT via the TGF‐β/Smads signalling pathway

**FIGURE 6:**
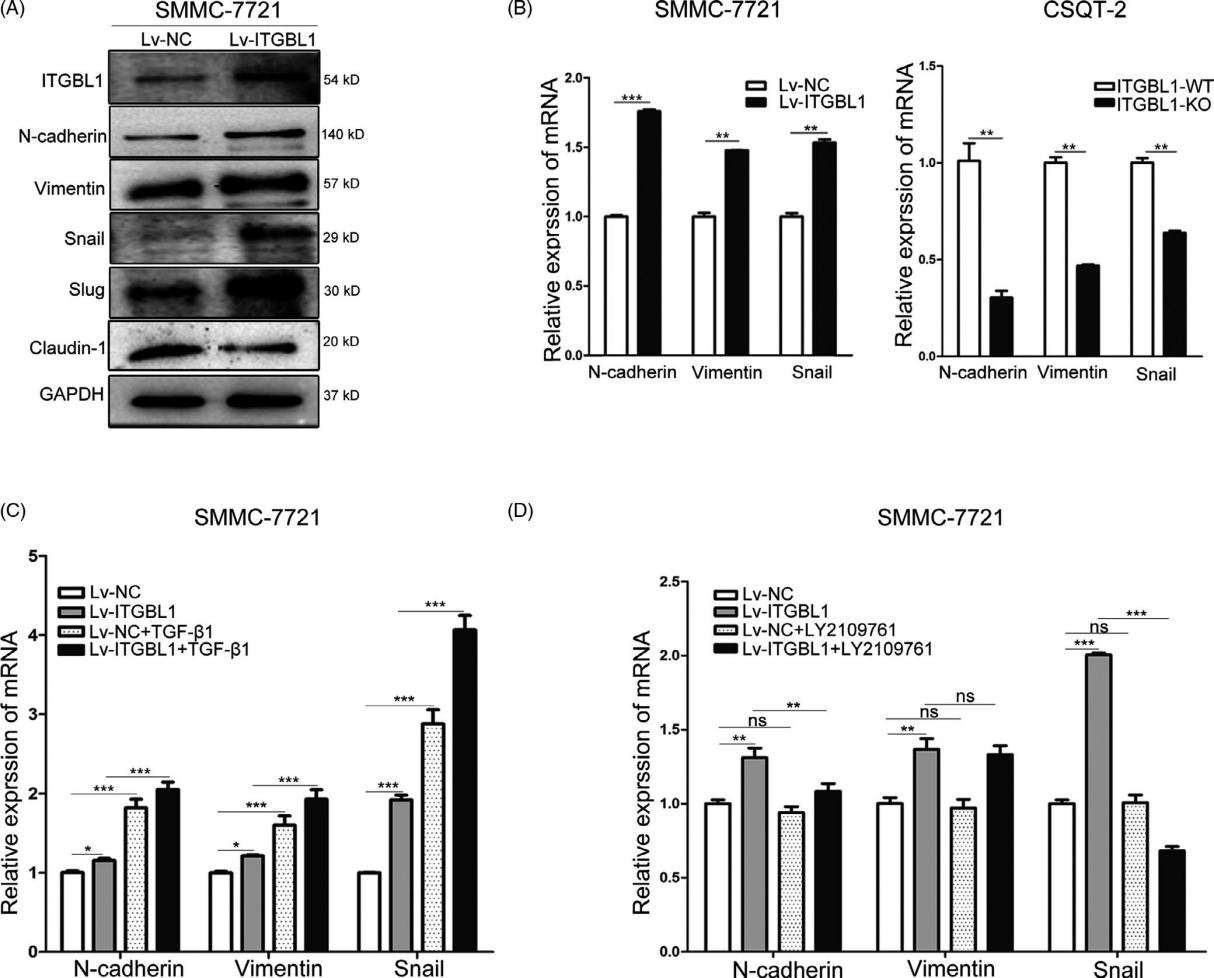
ITGBL1 promotes the epithelial‐mesenchymal transition in HCC cells. A, Western blotting analysis of EMT‐related genes in SMMC‐7721 cells with or without ITGBL1 overexpression. B, RT‐PCR analysis the EMT‐related genes in SMMC‐7721 cells with ITGBL1 overexpression (left) and CSQT‐2 cells with ITGBL1 knockout (right). Data are shown as the mean ± SD. C, RT‐PCR analysis for the quantification of EMT‐related genes in SMMC‐7721 cells with or without ITGBL1 overexpression following the treatment with TGF‐β1. Data are shown as the mean ± SD. D, RT‐PCR analysis for the quantification of the expression of EMT‐related genes in SMMC‐7721 cells with or without ITGBL1 overexpression following the treatment with the TGFBR1/2 dual inhibitor LY2109761. Data are shown as the mean ± SD. ^*^ *P* < .05, ^**^ *P* < .01 and ^***^ *P* < .01 indicate a significant difference between the groups

It has been reported that KRT17 is a downstream factor of TGF‐β1 and that ITGBL1 may stimulate liver fibrosis through regulating the TGF‐β1 signalling pathways.^12^ We determined whether ITGBL1 regulated the KRT17 expression through TGF‐β1 signalling pathway, which also induces the epithelial‐mesenchymal transition (EMT) in various types of cancers.^20^ The Western blotting results suggested that the expression of mesenchymal markers including N‐cadherin, Vimentin, Snail and Slug were significantly increased, while the epithelial marker Claudin‐1 were significantly reduced in ITGBL1‐overexpressing SMMC‐7721 cells (Figure 6A). The RT‐PCR results also confirmed that ITGBL1 overexpression increased the expression of N‐cadherin, Vimentin and Snail in SMMC‐7721 cells. In contrast, ITGBL1 knockout reduced the expression of N‐cadherin, Vimentin and Snail in CSQT‐2 cells (Figure 6B). Furthermore, we intended to investigate the mediatory roles of TGF‐β1 in ITGBL1‐induced EMT. Under the TGF‐β1 treatment, the mRNA levels of EMT‐related genes increased significantly (Figure 6C). Meanwhile, in SMMC‐7721 cells treated with LY2109761, a selective TGF‐β receptor type I/II (TGFBRI/II) dual inhibitor, the expression levels of these genes were significantly reduced (Figure 6D). These results indicated that TGF‐β1 may mediate the ITGBL1‐induced EMT in liver cancer cells.

**FIGURE 7:**
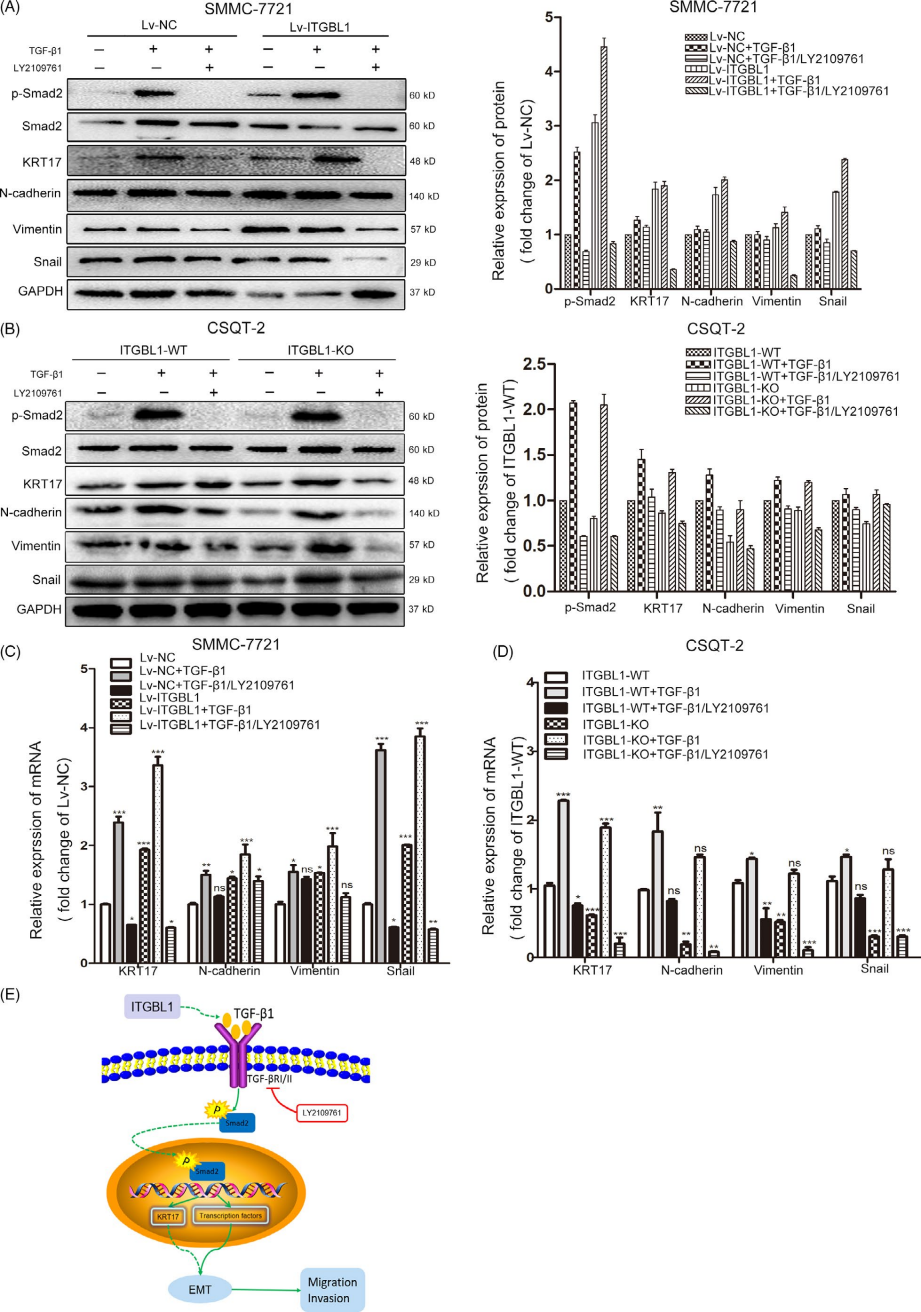
ITGBL1 promotes EMT via the TGF‐β/Smads signalling pathway. A, Western blotting analysis (left) and the greyscale analysis (right) of genes involved in EMT and downstream of the TGF‐β signalling pathway in SMMC‐7721 cells (with or without ITGBL1 overexpression) individually treated with TGF‐β1 or LY2109761 or the combination of TGF‐β1 and LY2109761 treatment. GAPDH was used as the internal loading control. B, Western blotting analysis (left) and the greyscale analysis (right) of EMT‐related genes and the genes downstream of the TGF‐β signalling pathway in CSQT‐2 cells (with or without ITGBL1 knockout) individually treated with TGF‐β1 or LY2109761 or the combination of TGF‐β1 and LY2109761 treatment. GAPDH was used as the internal loading control. C, RT‐PCR analysis for the quantification of the mRNA levels of EMT‐related genes and KRT17 in SMMC‐7721 cells with or without ITGBL1 overexpression following the treatment with TGF‐β1, LY2109761, or a combination of TGF‐β1 and LY2109761. Data are shown as the mean ± SD. D, RT‐PCR analysis for the quantification of the mRNA levels of EMT‐related genes and KRT17 in CSQT‐2 cells with or without ITGBL1 knockout following the treatment with TGF‐β1, LY2109761, or a combination of TGF‐β1 and LY2109761. Data are shown as the mean ± SD. E, A schematic model depicting that ITGBL1 stimulates the expression of KRT17 and induces EMT via TGF‐β/Smads signalling pathway, which promotes invasion and metastasis of HCC cells. ^*^ *P* < .05, ^**^ *P* < .01 and ^***^ *P* < .001 indicate a significant difference between the groups

To further explore whether ITGBL1 regulates KRT17 and EMT via the TGF‐β1/Smads signalling pathway, HCC cells with or without ITGBL1 overexpression or knockout were treated with 10 ng/mL TGF‐β1 and/or 1 µM LY2109761 inhibitor for 24 hours. As shown in Figure 7A, ITGBL1 overexpression enhanced the expression of p‐Smad2, KRT17, N‐cadherin, Vimentin and Snail in SMMC‐7721 cells. Meanwhile, the expression of p‐Smad2, KRT17, N‐cadherin, Vimentin and Snail increased significantly in SMMC‐7721 cells stimulated with TGF‐β1, but significantly decreased in SMMC‐7721 cells treated with TGF‐β1 and LY2109761 inhibitor (Figure 7A). Similar results were observed at the mRNA level as determined by the RT‐PCR assays (Figure 7C). In contrast, the expression of p‐Smad2, KRT17, N‐cadherin, Vimentin and Snail was reduced in ITGBL1‐knockout CSQT‐2 cells, compared to the wild‐type CSQT‐2 cells (Figure 7B); furthermore, the protein and mRNA expression levels of p‐Smad2, KRT17, N‐cadherin, Vimentin and Snail were reduced in CSQT‐2 cells treated with LY2109761 inhibitor (Figure 7B,D). These results suggested that ITGBL1 stimulates the expression of KRT17 and EMT via the TGF‐β1/Smads signalling pathway in HCC cells.

## 4 DISCUSSION

In clinical settings, intrahepatic and extrahepatic metastasis of HCC cells presents a major barrier for favourable clinical outcomes; however, the mechanisms underlying the tumour recurrence and metastasis remain elusive. Although emerging studies have unveiled the important roles of ITGBL1 in the invasion, migration and chemoresistance of malignant tumours,^8^, ^9^, ^21^, ^22^, ^23^, ^24^, ^25^, ^26^, ^27^ the roles of ITGBL1 in the development and progression of HCC are not clear. In this study, we showed that the expression of ITGBL1 in HCC tissues is increased compared to adjacent normal tissues, and ITGBL1 higher expression is correlated with incomplete tumour encapsulation and poorer OS in HCC patients. ITGBL1 could promote the cellular migration and invasion, which may be partially caused by the increased KRT17 expression level. In further, we demonstrated that ITGBL1 promotes the expression of KRT17 and other EMT‐related biomarkers through stimulating the TGF‐β1/Smads signalling pathway in HCC cells (Figure 7E). These results suggested that ITGBL1 might serve as a novel prognostic biomarker for HCC patients, and the ITGBL1/TGF‐β1/Smads signalling pathways could act as potential targets for metastasis prevention in HCC patients.

Previous studies have reported that the increased expression of ITGBL1 is associated with tumorigenesis and poorer prognosis in a variety of cancers. In ovarian cancer, ITGBL1 was upregulated in ovarian cancer tissues compared to adjacent non‐cancer tissues, and it was positively correlated with lymph node invasion and advanced FIGO stage.^8^, ^22^ In prostate cancer (PCa) patients, ITGBL1 was significantly upregulated and positively associated with lymph node metastasis status.^26^ In the current study, we found both the protein and mRNA levels of ITGBL1 were increased in HCC tumour tissues, compared with the adjacent non‐tumour tissues. In clinic, higher ITGBL1 was correlated with incomplete encapsulation of HCC patients and it was independently associated with the overall survival of HCC patients. Previous studies suggested that unencapsulated or incomplete encapsulated tumours could invade directly into the surrounding liver parenchyma, cause the destruction of the extracellular matrix and then migrate into the circulation.^28^ These indicated that ITGBL1 acts as an oncogene in HCC and may promote the migration and invasion of HCC cells. Li *et al* reported that breast cancer patients with higher ITGBL1 expression showed a greater degree of bone metastasis and bone‐only metastasis than those with lower ITGBL1 expression.^8^ ITGBL1 has also been shown to promote the migration, invasion and adhesion of non–small‐cell lung cancer, colorectal cancer and ovarian cancer cells.^9^, ^21^, ^27^ With the in vitro and in vivo models, we found that ITGBL1 overexpression promotes HCC cells migration and invasion, while ITGBL1 knockout inhibits the migration and invasion of HCC cells. These results suggested ITGBL1 may promote the disease progression and serve as novel predictive biomarker of the metastasis for various types of cancer.

Epithelial‐mesenchymal transition (EMT) has been recognized as an indispensable stage for cancer cell metastasis, and it plays a crucial role in the progression of various types of cancer.^29^, ^30^ Previous studies have demonstrated that EMT is regulated by several signalling pathways, such as the TGF‐β, hepatocyte growth factor (HGF), Wnt/β‐catenin, bone morphogenic protein (BMP), epidermal growth factor receptor, fibroblast growth factor and Notch signalling pathways.^31^ Among these, the TGF‐β signalling pathway per se is involved in a range of biological processes, including the inhibition of cell growth, cell migration and invasion, ECM remodelling and immune‐suppression, which was found to play important roles in EMT of multiple cancers EMT of multiple cancers.^20^, ^32^ TGF‐β1 plays key roles in modulating HCC aggressiveness through triggering the EMT of the cancer cells.^33^ Through gene‐set enrichment analysis (GSEA), Matsuyama et al^25^ have found that the expression of ITGBL1 may serve as an important indicator of an EMT phenotype in CRC. In PCa cells, upregulating ITGBL1 could enhance the invasion and migration abilities of PCs cells and promote the EMT.^26^ In this study, we found that the expression levels of EMT‐related biomarkers including Snail, Slug, Vimentin and N‐cadherin were increased following ITGBL1 overexpression. Considering the positive correlation between the expression levels of ITGBL1 and TGF‐β1 in our previous study,^12^ we speculated that TGF‐β signalling mediated the ITGBL1‐induced EMT. Following the stimulation of ITGBL1‐overexpressing SMMC‐7721 cells with TGF‐β1, we observed that the expression levels of p‐Smad2, KRT17, N‐cadherin, Vimentin and Snail increased, but were reduced in the SMMC‐7721 cells treated with the selective TGFBRI/II dual inhibitor LY2109761. These results suggested that ITGBL1 could promote EMT through activating TGF‐β/Smads signalling pathway.

Furthermore, in order to elucidate the underlying mechanisms responsible for the abnormal expression of ITGBL1, we performed the RNA‐sequencing analysis and found that KRT17 was one of the down streaming genes regulated by ITGBL1. KRT17, also known as cytokeratin 17, is a member of the keratin family of proteins. Previous studies have demonstrated that KRT17 could promote the progression of various malignancies, such as breast cancer,^34^ cervical carcinoma,^35^, ^36^, ^37^ gastric cancer,^38^ non–small‐cell lung cancer^39^ and skin squamous carcinoma.^40^ As a downstream factor of the TGF‐β1‐/ERK1/2‐/MZF1 signalling pathway, KRT17 has been shown to promote metastasis through enabling the acquisition of tumour microenvironment related cancer stem cell (CSC) properties in cervical cancer.^41^ A recent study reported that KRT17 could promote cell invasion and metastasis and EMT through AKT signalling pathway in oesophageal squamous cell carcinoma (ESCC).^42^ However, the expression of KRT17 and its correlation with ITGBL1 in HCC remains unclear. From the TCGA database, we found that the mRNA expression level of KRT17 is higher in primary liver tumour tissues compared to that in normal liver tissues. Besides, we found that the expression of KRT17 increased significantly in ITGBL1‐overexpressing SMMC‐7721 cells and decreased in ITGBL1‐knockout CSQT‐2 cells. Inhibition of the TGFBR1/II significantly inhibits the ITGBL1 induced expression of KRT17. These results indicated that KRT17 was regulated by ITGBL1 through its regulation on TGF‐β/Smads signalling pathway in HCC cells; however, the roles of KRT17 in HCC pathogenesis and progression remain to be elucidated in future.

In summary, our study showed that ITGBL1 was upregulated in HCC and associated with the OS in HCC patients. In HCC cells, ITGBL1 induces increased expression of KRT17 and EMT‐related genes through activating the TGF‐β/Smads signalling pathway, thereby promoting the migration and invasion of HCC cells. These data provided evidence for ITGBL1 as a promising prognostic factor for HCC, and it may also serve as a novel therapeutic target in HCC.

## CONFLICT OF INTEREST

The authors declare that they have no competing interests.

## AUTHOR CONTRIBUTIONS

WH performed the experiments. DY, MW, D.W and JC analysed the data. JL, Y.H, and BL designed the project. W.H and PC analysed the data and wrote the manuscript. PC and XZ designed the project and revised the manuscript.

## Supporting information

Fig S1

Table S1

Table S2

Table S3

Table S4

## Data Availability

The data that support the findings of this study are available from the corresponding author upon reasonable request.
