# Supplementary figures and images for: ITGBL1 promotes cell migration and invasion through stimulating the TGF‐β signalling pathway in hepatocellular carcinoma
Source: Cell Prolif. 2020 Jun 14;53(7):e12836. doi: 10.1111/cpr.12836 (PMC7377936; doi:10.1111/cpr.12836)

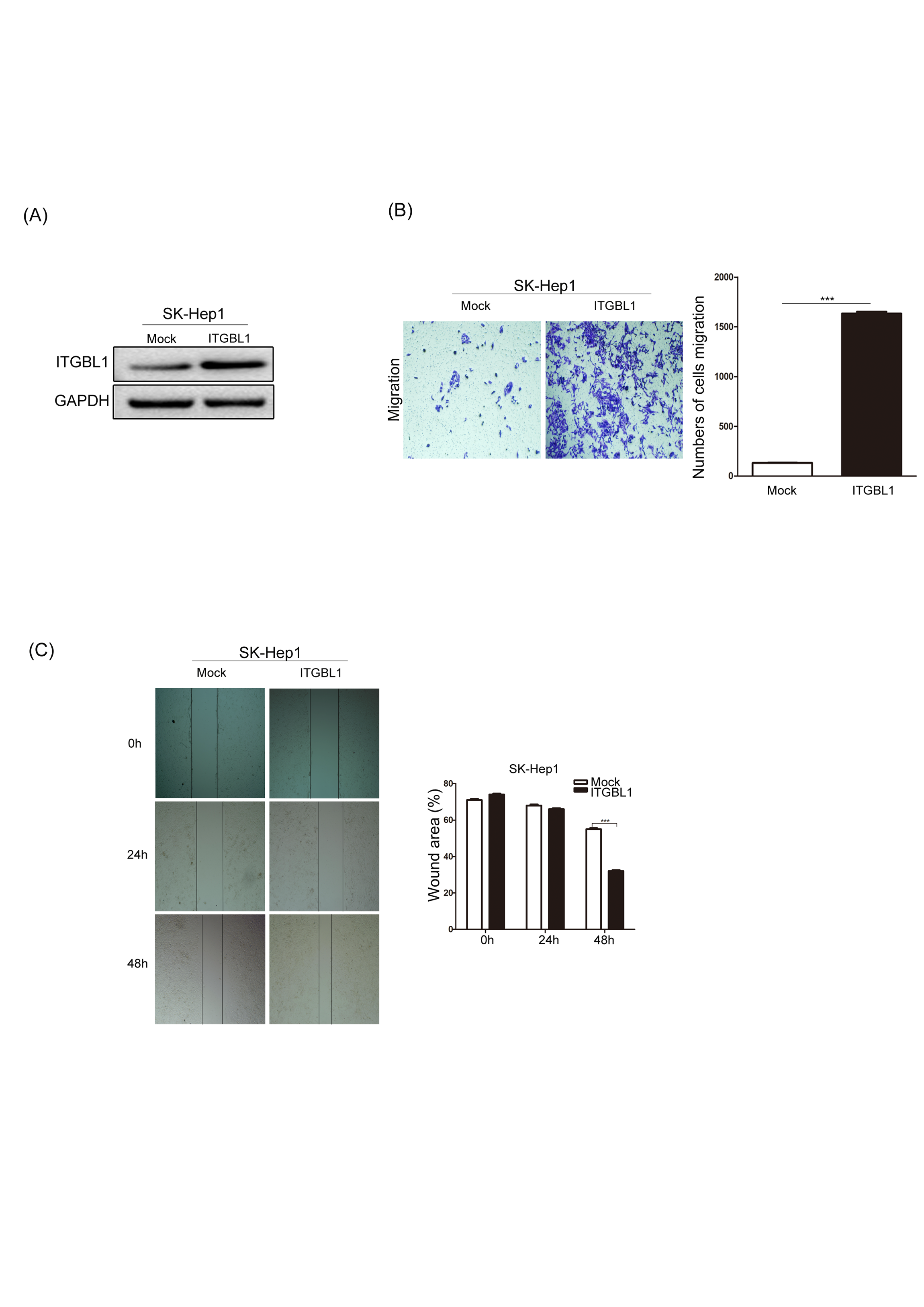

Supplement: Supplementary file 1 — Fig S1 [file CPR-53-e12836-s001.tif]
