# Supplementary material for: ITGBL1 promotes cell migration and invasion through stimulating the TGF‐β signalling pathway in hepatocellular carcinoma
Source: Cell Prolif. 2020 Jun 14;53(7):e12836. doi: 10.1111/cpr.12836 (PMC7377936; doi:10.1111/cpr.12836)
Supplement: Supplementary file 2 — Table S1 [file CPR-53-e12836-s002.docx]

| **Characteristics** | **No. of case** | **ITGBL1** | | |
| --- | --- | --- | --- | --- |
|  |  | **Low** | **High** | **P** |
| Gender |  |  |  |  |
| Male | 80 | 31 | 49 | 1.000 |
| Female | 18 | 7 | 11 |  |
| Age(years) |  |  |  |  |
| ＜50 | 44 | 18 | 26 | 0.835 |
| ≥50 | 54 | 20 | 34 |  |
| HbsAg |  |  |  |  |
| Negative | 12 | 2 | 10 | 0.126 |
| Positive | 86 | 35 | 51 |  |
| AFP,ng/ml |  |  |  |  |
| ＜400 | 37 | 13 | 24 | 0.670 |
| ≥400 | 61 | 25 | 36 |  |
| Cirrhosis |  |  |  |  |
| Negative | 56 | 20 | 36 | 0.533 |
| Positive | 42 | 18 | 24 |  |
| Tumor size, cm |  |  |  |  |
| ＜5 | 49 | 20 | 29 | 0.836 |
| ≥5 | 49 | 18 | 31 |  |
| Tumor number |  |  |  |  |
| Singer | 87 | 36 | 51 | 0.194 |
| Multiple | 11 | 2 | 9 |  |
| Tumor differentiation |  |  |  |  |
| Ⅰ-Ⅱ | 14 | 5 | 9 | 1.000 |
| Ⅲ-Ⅳ | 84 | 33 | 51 |  |
| Tumor encapsulation |  |  |  |  |
| Intact | 45 | 12 | 33 | 0.037* |
| Non-intact | 53 | 26 | 27 |  |
| Vascular invasion |  |  |  |  |
| Negative | 76 | 29 | 47 | 0.809 |
| Positive | 22 | 9 | 13 |  |

**Supplement Table 1.** Correlation of ITGBL1 expression with clinicopatholoical factors in 98 HCC patients.

Statistical analyses were performed by the Pearson’s χ2 test

*P < 0.05.

**P < 0.01.
