# Supplementary material for: ITGBL1 promotes cell migration and invasion through stimulating the TGF‐β signalling pathway in hepatocellular carcinoma
Source: Cell Prolif. 2020 Jun 14;53(7):e12836. doi: 10.1111/cpr.12836 (PMC7377936; doi:10.1111/cpr.12836)
Supplement: Supplementary file 3 — Table S2 [file CPR-53-e12836-s003.docx]

**Supplement Table 2.** Primers used for sgRNA synthesis and sequencing.

| Primer names |  | | Sequences | |
| --- | --- | --- | --- | --- |
| ITGBL1 sgRNA 1 | | Forward | | CACCGTCCAGTGCGATATCACCCCC |
| ITGBL1 sgRNA 1 | | Reverse | | AAACGGGGGTGATATCGCACTGGAC |
| ITGBL1 sgRNA 2 | | Forward | | CACCGCGAAGATGCACGTCTCCAGA |
| ITGBL1 sgRNA 2 | | Reverse | | AAACTCTGGAGACGTGCATCTTCGC |
| ITGBL1 sgRNA 3 | | Forward | | CACCGGGTTTAGGTACATGTCACTG |
| ITGBL1 sgRNA 3 | | Reverse | | AAACCAGTGACATGTACCTAAACCC |
| ITGBL1 1# | | Forward | | TGATGTGTTTGGAGAAAGCTGT |
| ITGBL1 1# | | Reverse | | AGTGAAGCTTAAAGAATGACAGC |
| ITGBL1 2# | | Forward | | AGGAATGTGGAGCTGTGACA |
| ITGBL1 2# | | Reverse | | GGAGGGCTGCCAAAGAAATT |
| ITGBL1 3# | | Forward | | TGCATTGGTTGGATGGCTAT |
| ITGBL1 3# | | Reverse | | TGCCATAGTCTCACTCTCCT |
