# Supplementary material for: ITGBL1 promotes cell migration and invasion through stimulating the TGF‐β signalling pathway in hepatocellular carcinoma
Source: Cell Prolif. 2020 Jun 14;53(7):e12836. doi: 10.1111/cpr.12836 (PMC7377936; doi:10.1111/cpr.12836)
Supplement: Supplementary file 4 — Table S3 [file CPR-53-e12836-s004.docx]

**Supplement Table 3.** Primers used for real-time PCR.

| Primer names |  | Sequences |
| --- | --- | --- |

| ITGBL1 | Forward | TGCATTGGTTGGATGGCTAT |
| --- | --- | --- |
| ITGBL1 | Reverse | TGCCATAGTCTCACTCTCCT |
| GAPDH | Forward | TGCACCACCAACTGCTTAGC |
| GAPDH | Reverse | GGCATGGACTGTGGTCATGAG |
| N-cadherin | Forward | CCATCAAGCCTGTGGGAATC |
| N-cadherin | Reverse | GCAGATCGGACCGGATACTG |
| Vimentin | Forward | ACACCCTGCAATCTTTCAGACA |
| Vimentin | Reverse | GATTCCACTTTGCGTTCAAGGT |
| Snail | Forward | CGGAAGCCCAACTATAGCGA |
| Snail | Reverse | AGAGTCCCAGATGAGGGTGG |
| MMP17 | Forward | CACCAAGTGGAACAAGAGGA |
| MMP17 | Reverse | AGACACAGACTCCCGCACAC |
| KRT17 | Forward | GGTGGGTGGTGAGATCAATGT |
| KRT17 | Reverse | CGCGGTTCAGTTCCTCTGTC |
| SMOC1 | Forward | GCTACCAGGCTGTCCAGAAG |
| SMOC1 | Reverse | GTTGCTGCTATTGCTGTCCA |
| WNT6 | Forward | GGTTATGGACCCTACCAGCA |
| WNT6 | Reverse | AACTGGAACTGGCACTCTCG |
| FOXQ1 | Forward | CGACTGCTTCGTCAAGGT |
| FOXQ1 | Reverse | CCGTCGGCGAAGGTGTA |
| VEGFA | Forward | GCCTTGCTGCTCTACCTCCA |
| VEGFA | Reverse | CAAGGCCCACAGGGATTTT |
| CA9 | Forward | GAAAGGCTGTTCGACAGAAGGT |
| CA9 | Reverse | GCTCTTTCAAAGAATGAGGCAACT |
| FOS | Forward | GAGAGCTGGTAGTTAGTAGCATGTTGA |
| FOS | Reverse | AATTCCAATAATGAACCCAATAGATTAGTTA |
