# Supplementary material for: ITGBL1 promotes cell migration and invasion through stimulating the TGF‐β signalling pathway in hepatocellular carcinoma
Source: Cell Prolif. 2020 Jun 14;53(7):e12836. doi: 10.1111/cpr.12836 (PMC7377936; doi:10.1111/cpr.12836)
Supplement: Supplementary file 5 — Table S4 [file CPR-53-e12836-s005.docx]

**Supplement Table 4.** The downstream differentially expressed genes of ITGBL1 in SMMC-7721 cells.

| **Ensembl ID** | **Gene** | **logFC** | **t** | **Adjusted P-Value** |
| --- | --- | --- | --- | --- |
| ENSG00000198542 | ITGBL1 | 4.82068695 | 56.06247 | 1.53E-14 |
| ENSG00000103888 | KIAA1199 | 2.88208383 | 34.29088 | 2.84E-12 |
| ENSG00000243137 | PSG4 | -1.5790344 | -19.0592 | 1.37E-09 |
| ENSG00000172379 | ARNT2 | 1.45496942 | 18.86266 | 1.52E-09 |
| ENSG00000128510 | CPA4 | -1.5615379 | -17.7768 | 2.82E-09 |
| ENSG00000059804 | SLC2A3 | 2.08437595 | 14.422 | 2.42E-08 |
| ENSG00000242265 | PEG10 | -1.1555422 | -14.3277 | 2.59E-08 |
| ENSG00000180537 | RNF182 | 1.16497967 | 13.8373 | 3.69E-08 |
| ENSG00000160179 | ABCG1 | 1.16616723 | 13.77458 | 3.86E-08 |
| ENSG00000136379 | ABHD17C | 0.98574492 | 12.63942 | 9.22E-08 |
| ENSG00000176658 | MYO1D | 1.04016894 | 12.19065 | 1.33E-07 |
| ENSG00000204103 | MAFB | 0.98675197 | 11.73606 | 1.94E-07 |
| ENSG00000250208 | FZD10-AS1 | 0.94307926 | 11.66664 | 2.06E-07 |
| ENSG00000112972 | HMGCS1 | -0.9096781 | -11.5458 | 2.28E-07 |
| ENSG00000105141 | CASP14 | 1.23935444 | 11.52045 | 2.33E-07 |
| ENSG00000128422 | KRT17 | 1.12473752 | 11.24415 | 2.97E-07 |
| ENSG00000170873 | MSS1 | 0.96135396 | 11.2046 | 3.07E-07 |
| ENSG00000111348 | ARHGDIB | -0.9708413 | -11.038 | 3.56E-07 |
| ENSG00000175445 | LPL | -1.1603568 | -10.9444 | 3.88E-07 |
| ENSG00000270526 | SNRPGP19 | 1.04168993 | 10.69846 | 4.85E-07 |
| ENSG00000260604 | RP1-140K8.5 | -0.935601 | -10.4498 | 6.11E-07 |
| ENSG00000149948 | HMGA2 | -0.9053403 | -10.2934 | 7.08E-07 |
| ENSG00000148677 | ANKRD1 | -0.9432127 | -10.1778 | 7.91E-07 |
| ENSG00000196557 | CACNA1H | 0.97944185 | 10.13739 | 8.22E-07 |
| ENSG00000214049 | UCA1 | -1.0459042 | -10.0221 | 9.19E-07 |
| ENSG00000159399 | HK2 | 0.8812477 | 10.01214 | 9.28E-07 |
| ENSG00000204941 | PSG5 | -1.0935217 | -9.6701 | 1.30E-06 |
| ENSG00000104549 | SQLE | -0.7247407 | -9.59609 | 1.40E-06 |
| ENSG00000174827 | PDZK1 | -0.941916 | -9.5946 | 1.40E-06 |
| ENSG00000073792 | IGF2BP2 | -0.748519 | -9.54578 | 1.47E-06 |
| ENSG00000182885 | GPR97 | 0.71856832 | 9.536405 | 1.49E-06 |
| ENSG00000135678 | CPM | 0.72192042 | 9.329747 | 1.84E-06 |
| ENSG00000198732 | SMOC1 | 0.70520044 | 9.176766 | 2.15E-06 |
| ENSG00000198796 | ALPK2 | -0.7111982 | -9.06749 | 2.41E-06 |
| ENSG00000268388 | FENDRR | 0.74763076 | 8.95405 | 2.72E-06 |
| ENSG00000143847 | PPFIA4 | 1.05502633 | 8.684963 | 3.63E-06 |
| ENSG00000112319 | EYA4 | -0.6970803 | -8.63127 | 3.85E-06 |
| ENSG00000165029 | ABCA1 | 0.68606475 | 8.617237 | 3.91E-06 |
| ENSG00000153071 | DAB2 | -0.7219405 | -8.47923 | 4.56E-06 |
| ENSG00000111432 | FZD10 | 1.11788933 | 8.464013 | 4.63E-06 |
| ENSG00000127990 | SGCE | -0.7921916 | -8.46399 | 4.63E-06 |
| ENSG00000227619 | RP11-492E3.2 | 1.01265938 | 8.453869 | 4.69E-06 |
| ENSG00000167281 | RBFOX3 | 0.69744141 | 8.395329 | 5.00E-06 |
| ENSG00000057657 | PRDM1 | 0.676976 | 8.391403 | 5.03E-06 |
| ENSG00000128165 | ADM2 | 0.80835232 | 8.378643 | 5.10E-06 |
| ENSG00000082074 | FYB | -0.6841446 | -8.34104 | 5.32E-06 |
| ENSG00000186832 | KRT16 | 0.72188088 | 8.253058 | 5.87E-06 |
| ENSG00000173698 | GPR64 | -0.628978 | -8.22458 | 6.06E-06 |
| ENSG00000050405 | LIMA1 | -0.8184798 | -8.17396 | 6.42E-06 |
| ENSG00000138356 | AOX1 | -0.6470199 | -8.16354 | 6.50E-06 |
| ENSG00000138347 | MYPN | -0.6114713 | -8.16354 | 6.50E-06 |
| ENSG00000170345 | FOS | 0.73931585 | 8.132501 | 6.74E-06 |
| ENSG00000104419 | NDRG1 | 1.35881908 | 8.06455 | 7.28E-06 |
| ENSG00000123977 | DAW1 | -0.6631203 | -7.98254 | 8.01E-06 |
| ENSG00000099194 | SCD | 0.61212656 | 7.979931 | 8.03E-06 |
| ENSG00000163083 | INHBB | 0.6758319 | 7.960538 | 8.21E-06 |
| ENSG00000122861 | PLAU | -0.651036 | -7.76364 | 1.04E-05 |
| ENSG00000147689 | FAM83A | 0.79286014 | 7.731985 | 1.07E-05 |
| ENSG00000108448 | TRIM16L | -0.5859164 | -7.7315 | 1.08E-05 |
| ENSG00000215146 | RP11-313J2.1 | -0.6221677 | -7.71216 | 1.10E-05 |
| ENSG00000168209 | DDIT4 | 0.67789515 | 7.698547 | 1.12E-05 |
| ENSG00000107159 | CA9 | 1.14032759 | 7.627547 | 1.22E-05 |
| ENSG00000052802 | MSMO1 | -0.7727319 | -7.60932 | 1.24E-05 |
| ENSG00000137033 | IL33 | 0.71756635 | 7.578251 | 1.29E-05 |
| ENSG00000116852 | KIF21B | 0.83076429 | 7.473208 | 1.47E-05 |
| ENSG00000125538 | IL1B | 0.61634306 | 7.472645 | 1.47E-05 |
| ENSG00000102312 | PORCN | 0.60931302 | 7.441339 | 1.53E-05 |
| ENSG00000169252 | ADRB2 | -0.6138548 | -7.37509 | 1.65E-05 |
| ENSG00000062282 | DGAT2 | 0.61598079 | 7.367154 | 1.67E-05 |
| ENSG00000167772 | ANGPTL4 | 0.75708224 | 7.340066 | 1.73E-05 |
| ENSG00000164488 | DACT2 | 0.92925699 | 7.300205 | 1.81E-05 |
| ENSG00000196730 | DAPK1 | -0.6869543 | -7.27866 | 1.86E-05 |
| ENSG00000105877 | DNAH11 | -0.6007864 | -7.27324 | 1.88E-05 |
| ENSG00000061656 | SPAG4 | 0.76794684 | 7.26724 | 1.89E-05 |
| ENSG00000224321 | RP11-169K16.6 | -0.6030442 | -7.26379 | 1.90E-05 |
| ENSG00000129270 | MMP28 | 0.81758635 | 7.24772 | 1.94E-05 |
| ENSG00000112175 | BMP5 | -0.6728845 | -7.18404 | 2.10E-05 |
| ENSG00000075223 | SEMA3C | -0.6342945 | -7.04974 | 2.48E-05 |
| ENSG00000164509 | IL31RA | -0.5954143 | -7.02874 | 2.55E-05 |
| ENSG00000234745 | HLA-B | 0.80747613 | 6.98536 | 2.69E-05 |
| ENSG00000112715 | VEGFA | 0.66192316 | 6.979837 | 2.71E-05 |
| ENSG00000175745 | NR2F1 | -0.6060752 | -6.96953 | 2.75E-05 |
| ENSG00000125746 | EML2 | 0.59151065 | 6.965353 | 2.76E-05 |
| ENSG00000133477 | FAM83F | 0.59337131 | 6.937299 | 2.87E-05 |
| ENSG00000117525 | F3 | -0.7191678 | -6.92597 | 2.91E-05 |
| ENSG00000137962 | ARHGAP29 | -0.623367 | -6.88276 | 3.07E-05 |
| ENSG00000128591 | FLNC | -1.1954615 | -6.83514 | 3.27E-05 |
| ENSG00000231924 | PSG1 | -0.7048279 | -6.76562 | 3.58E-05 |
| ENSG00000185745 | IFIT1 | -0.6205011 | -6.74915 | 3.65E-05 |
| ENSG00000130720 | FIBCD1 | 0.75333366 | 6.627636 | 4.29E-05 |
| ENSG00000129354 | AP1M2 | -0.6536395 | -6.618 | 4.34E-05 |
| ENSG00000162729 | IGSF8 | 0.72086253 | 6.600165 | 4.45E-05 |
| ENSG00000186765 | FSCN2 | 0.69668767 | 6.578071 | 4.58E-05 |
| ENSG00000115363 | EVA1A | -0.6111823 | -6.56715 | 4.64E-05 |
| ENSG00000173918 | C1QTNF1 | 0.62473973 | 6.536882 | 4.84E-05 |
| ENSG00000146674 | IGFBP3 | 1.42213768 | 6.524299 | 4.92E-05 |
| ENSG00000101188 | NTSR1 | 1.03297006 | 6.479082 | 5.22E-05 |
| ENSG00000081923 | ATP8B1 | -0.6771392 | -6.4639 | 5.33E-05 |
| ENSG00000125772 | GPCPD1 | 0.60991421 | 6.439976 | 5.50E-05 |
| ENSG00000124882 | EREG | 0.60675842 | 6.375337 | 6.01E-05 |
| ENSG00000049239 | H6PD | 0.71043938 | 6.363179 | 6.11E-05 |
| ENSG00000111057 | KRT18 | -0.6292672 | -6.32833 | 6.40E-05 |
| ENSG00000184500 | PROS1 | -0.6271441 | -6.3105 | 6.56E-05 |
| ENSG00000060656 | PTPRU | 1.07581197 | 6.309245 | 6.57E-05 |
| ENSG00000167191 | GPRC5B | -0.6781813 | -6.26217 | 7.01E-05 |
| ENSG00000136286 | MYO1G | 0.73018032 | 6.218561 | 7.44E-05 |
| ENSG00000108932 | SLC16A6 | 0.77700116 | 6.214267 | 7.48E-05 |
| ENSG00000219607 | PPP1R3G | 0.63456002 | 6.204739 | 7.58E-05 |
| ENSG00000187474 | FPR3 | -0.6028623 | -6.13316 | 8.37E-05 |
| ENSG00000214145 | LINC00887 | 0.83176255 | 6.132493 | 8.38E-05 |
| ENSG00000162591 | MEGF6 | 1.00064397 | 6.095033 | 8.82E-05 |
| ENSG00000111275 | ALDH2 | -0.6482409 | -6.05789 | 9.29E-05 |
| ENSG00000145287 | PLAC8 | -0.703939 | -6.04448 | 9.47E-05 |
| ENSG00000128965 | CHAC1 | 0.5902913 | 6.029225 | 9.67E-05 |
| ENSG00000145569 | FAM105A | -0.5994881 | -6.0231 | 9.76E-05 |
| ENSG00000198753 | PLXNB3 | 0.8187343 | 5.96551 | 0.000105779 |
| ENSG00000164651 | SP8 | 0.7164514 | 5.946515 | 0.00010865 |
| ENSG00000067064 | IDI1 | -0.6892783 | -5.91377 | 0.000113797 |
| ENSG00000079308 | TNS1 | 0.70981426 | 5.867786 | 0.00012147 |
| ENSG00000164683 | HEY1 | 0.62033638 | 5.79896 | 0.000134005 |
| ENSG00000146242 | TPBG | 0.68638017 | 5.744821 | 0.000144835 |
| ENSG00000150782 | IL18 | -0.7407445 | -5.67758 | 0.000159603 |
| ENSG00000196517 | SLC6A9 | 0.80927464 | 5.613217 | 0.00017525 |
| ENSG00000123384 | LRP1 | 0.86238193 | 5.550767 | 0.000192005 |
| ENSG00000124466 | LYPD3 | 0.89598553 | 5.519928 | 0.000200901 |
| ENSG00000197632 | SERPINB2 | 0.68980857 | 5.517479 | 0.000201626 |
| ENSG00000169605 | GKN1 | -0.7717239 | -5.47011 | 0.000216213 |
| ENSG00000144152 | FBLN7 | 0.62739352 | 5.464605 | 0.000217979 |
| ENSG00000103241 | FOXF1 | 0.91657772 | 5.434306 | 0.000227982 |
| ENSG00000114268 | PFKFB4 | 0.67212822 | 5.366628 | 0.000252133 |
| ENSG00000131094 | C1QL1 | 1.16710189 | 5.197698 | 0.000325099 |
| ENSG00000116285 | ERRFI1 | 0.75616384 | 5.155605 | 0.000346574 |
| ENSG00000113083 | LOX | 0.96340422 | 5.123998 | 0.000363687 |
| ENSG00000132470 | ITGB4 | 0.85711826 | 5.062697 | 0.000399485 |
| ENSG00000108821 | COL1A1 | 0.69813525 | 4.959683 | 0.000468319 |
| ENSG00000213923 | CSNK1E | 0.59265975 | 4.947064 | 0.000477578 |
| ENSG00000105974 | CAV1 | -0.6031726 | -4.90537 | 0.000509574 |
| ENSG00000162878 | PKDCC | 0.7477245 | 4.870457 | 0.000538111 |
| ENSG00000198455 | ZXDB | 0.64743485 | 4.854732 | 0.000551511 |
| ENSG00000169583 | CLIC3 | -0.6780929 | -4.84229 | 0.000562369 |
| ENSG00000181458 | TMEM45A | 0.76508227 | 4.816163 | 0.000585898 |
| ENSG00000184961 | AL772307.1 | 0.63777613 | 4.800534 | 0.000600471 |
| ENSG00000107731 | UNC5B | 0.68454093 | 4.764559 | 0.00063549 |
| ENSG00000183092 | BEGAIN | 0.6710233 | 4.652582 | 0.000758993 |
| ENSG00000129521 | EGLN3 | 0.6317432 | 4.619265 | 0.000800449 |
| ENSG00000115596 | WNT6 | 0.64378293 | 4.531482 | 0.000921518 |
| ENSG00000101187 | SLCO4A1 | 0.60094643 | 4.495599 | 0.00097643 |
| ENSG00000204991 | SPIRE2 | 0.74651027 | 4.490484 | 0.000984534 |
| ENSG00000067840 | PDZD4 | 0.68834288 | 4.443918 | 0.001061646 |
| ENSG00000174938 | SEZ6L2 | 0.59805552 | 4.394324 | 0.001150795 |
| ENSG00000228589 | SPCS2P4 | 0.82069406 | 4.354594 | 0.001227877 |
| ENSG00000169184 | MN1 | 0.81443945 | 4.353117 | 0.001230844 |
| ENSG00000198598 | MMP17 | 0.8013434 | 4.350678 | 0.001235762 |
| ENSG00000162614 | NEXN | -0.6131695 | -4.34612 | 0.001245011 |
| ENSG00000143878 | RHOB | 0.59640224 | 4.287907 | 0.001369686 |
| ENSG00000185033 | SEMA4B | 0.6053007 | 4.264387 | 0.001423716 |
| ENSG00000256393 | AC138123.2 | -0.6064859 | -4.22344 | 0.001523175 |
| ENSG00000142173 | COL6A2 | 0.66968154 | 4.187223 | 0.001617205 |
| ENSG00000123358 | NR4A1 | 0.92530423 | 4.101805 | 0.001863858 |
| ENSG00000198444 | F8A2 | 0.87074955 | 4.018126 | 0.00214382 |
| ENSG00000130827 | PLXNA3 | 0.61288308 | 4.009891 | 0.002173649 |
| ENSG00000186907 | RTN4RL2 | 0.66199212 | 3.852275 | 0.002836096 |
| ENSG00000173599 | PC | 0.62044003 | 3.795103 | 0.003125595 |
| ENSG00000105520 | DKFZP761J1410 | 0.59456533 | 3.740862 | 0.003428656 |
| ENSG00000020181 | GPR124 | 0.70297509 | 3.72505 | 0.003522616 |
| ENSG00000198517 | MAFK | 0.71306302 | 3.695209 | 0.003707287 |
| ENSG00000171298 | GAA | 0.65373069 | 3.622094 | 0.004203325 |
| ENSG00000165390 | ANXA8 | 0.70721375 | 3.621269 | 0.004209297 |
| ENSG00000134590 | FAM127A | 0.60015511 | 3.549156 | 0.004766762 |
| ENSG00000074855 | ANO8 | 0.66535923 | 3.490351 | 0.005277432 |
| ENSG00000244257 | PKD1P1 | 0.62123731 | 3.440344 | 0.005755878 |
| ENSG00000167996 | FTH1 | -0.6136163 | -3.43817 | 0.005777623 |
| ENSG00000228594 | C1orf233 | 0.71369582 | 3.278746 | 0.007629355 |
| ENSG00000171282 | RP11-1055B8.7 | 0.65674846 | 3.168003 | 0.009263908 |
| ENSG00000090006 | LTBP4 | 0.67760737 | 3.16185 | 0.009364556 |
| ENSG00000072310 | SREBF1 | 0.6823832 | 3.140683 | 0.009719369 |
| ENSG00000107331 | ABCA2 | 0.65353909 | 3.037853 | 0.011647397 |
| ENSG00000169715 | M1E | -0.6751945 | -3.03764 | 0.011651825 |
| ENSG00000165655 | ZNF503 | 0.74421346 | 2.996338 | 0.012531662 |
| ENSG00000260075 | NSFP1 | 0.61543993 | 2.947851 | 0.013650633 |
| ENSG00000254667 | AP000783.1 | 0.67352784 | 2.907284 | 0.01466387 |
| ENSG00000168993 | CPLX1 | 0.68537431 | 2.894136 | 0.015008252 |
| ENSG00000008710 | PKD1 | 0.58942432 | 2.772239 | 0.018613395 |
| ENSG00000272060 | RNA18S5 | 0.92729324 | 2.767035 | 0.018785269 |
| ENSG00000164379 | FOXQ1 | 0.76312771 | 2.682994 | 0.021790298 |
| ENSG00000265150 | RN7SL2 | 0.58918322 | 2.645411 | 0.023284379 |
| ENSG00000129757 | CDKN1C | 0.66703593 | 2.549658 | 0.027564759 |
| ENSG00000265660 | MIR4664 | 0.66972811 | 2.548064 | 0.027642218 |
| ENSG00000183397 | C19orf71 | 0.78476336 | 2.514961 | 0.029300077 |
| ENSG00000258486 | RN7SL1 | 0.59912268 | 2.485563 | 0.030853992 |
| ENSG00000130881 | LRP3 | 0.59342494 | 2.397146 | 0.036029865 |
| ENSG00000132003 | ZSWIM4 | 0.59392095 | 2.396916 | 0.036044354 |
| ENSG00000065717 | TLE2 | 0.61728613 | 2.366779 | 0.037995698 |
| ENSG00000130675 | MNX1 | 0.61533485 | 2.351093 | 0.039051608 |
| ENSG00000185950 | IRS2 | 0.69447281 | 2.307786 | 0.04211657 |
| ENSG00000205572 | SERF1B | 0.77031977 | 2.259946 | 0.045772553 |
